# Outcomes of Drug‐Coated Balloon Versus Drug‐Eluting Stent for In‐Stent Restenosis and De‐Novo Lesions: A Meta‐Analysis of Randomized Controlled Trials

**DOI:** 10.1002/hsr2.72061

**Published:** 2026-04-24

**Authors:** Arman Soltani Moghadam, Sina Kazemian, Shayan Shojaei, Saman Soltani Moghadam, Reza Hosseini Dolama, Rasoul Ebrahimi, Fatemeh Ojaghi Shirmard, Seyed Morteza Ali Pourfaraji, Mohammad Sadeq Najafi, Sara Hobaby, Hamidreza Soleimani, Masih Tajdini, Behnam Tehrani, Dhaval Kolte, Islam Elgendy, Sandeep Basavarajaiah, Azeem Latib, Yasar Sattar, M. Chadi Alraies, Kaveh Hosseini

**Affiliations:** ^1^ Tehran Heart Center, Cardiovascular Diseases Research Institute, Tehran University of Medical Sciences, Tehran, Iran; ^2^ Division of Cardiology, Department of Medicine Johns Hopkins University School of Medicine Baltimore Maryland USA; ^3^ Inova Heart and Vascular Institute Falls Church Virginia USA; ^4^ Division of Cardiology Massachusetts General Hospital and Harvard Medical School Boston Massachusetts USA; ^5^ Division of Cardiovascular Medicine Gill Heart Institute, University of Kentucky Lexington Kentucky USA; ^6^ Department of Cardiology Heartlands Hospital, University Hospitals Birmingham Birmingham UK; ^7^ Division of Cardiology Montefiore Medical Center, The Bronx New York USA; ^8^ Department of Cardiology West Virginia University Morgantown West Virginia USA; ^9^ Department of Cardiology Detroit Medical Center Detroit Michigan USA; ^10^ Department of Cardiology Copenhagen University Hospital – Herlev and Gentofte Copenhagen Denmark

**Keywords:** drug‐coated balloon, drug‐eluting stent, in‐stent restenosis, percutaneous coronary intervention, small vessel disease, ST‐segment elevation myocardial infarction

## Abstract

**Background and Aims:**

Drug‐coated balloons (DCBs) and drug‐eluting stents (DESs) are commonly used in percutaneous coronary interventions (PCI), but their long‐term comparative effectiveness remains unclear. This meta‐analysis aimed to compare the clinical and angiographic outcomes of DCB versus DES in patients with in‐stent restenosis (ISR) and de novo lesions, including ST‐elevation myocardial infarction (STEMI) and small vessel disease (SVD).

**Methods:**

A systematic search identified randomized controlled trials (RCTs) comparing DCBs and DESs. The primary outcome was target lesion revascularization (TLR), with secondary outcomes including all‐cause mortality, cardiovascular mortality, myocardial infarction, stent/lesion thrombosis, and late lumen loss (LLL). The frequentist network meta‐analysis and binary random‐effect analysis were used to compare DCB versus. DES.

**Results:**

A total of 21 RCTs with 4244 patients were included. In ISR patients, the risk of TLR was comparable between DCB vs. DES at 1‐year (OR: 1.36, 95% CI: 0.86–2.14) and beyond 1‐year (OR: 1.23, 95% CI: 0.79–1.93). DCB and DES showed no significant difference in other secondary outcomes at 1‐year and beyond. In STEMI and SVD patients, DCB and DES demonstrated similar outcomes, except for a lower LLL after DCB in patients with SVD (SMD: −0.38, 95% CI: −0.53 to −0.22).

**Conclusion:**

DCB and DES were associated with a similar risk of TLR in patients with ISR, STEMI, and SVD. The results were consistent at 1‐year and beyond time intervals. Furthermore, DCB was associated with a lower LLL compared to DES in patients with SVD.

## Summary

- Drug‐coated balloons (DCBs) have emerged as a promising device option in percutaneous coronary interventions and have been recently given FDA approval.
- In this study, DCB demonstrated comparable clinical outcomes with drug‐eluting stent (DES) in patients with ISR and de novo lesions, including STEMI and SVD, except for lower LLL following DCB compared to DES in patients with SVD.
- Future trials are warranted to compare the clinical outcomes of DCB with DES in complex clinical settings like bifurcation or chronic total occlusion lesions.

**Abbreviations**
BMS: bare metal stent
DCB: drug‐coated balloon
DES: drug‐eluting stent
ISR: in‐stent restenosis
LLL: late lumen loss
PCI: percutaneous coronary intervention
RCT: randomized controlled trials
STEMI: ST‐elevation myocardial infarction
SVD: small vessel disease
TLR: target lesion revascularization

## 1 Introduction

The use of drug‐eluting stents (DES) is currently considered the treatment of choice in patients undergoing percutaneous coronary intervention (PCI) [1]. However, DES continues to be linked with target lesion failure during follow‐up, primarily due to device‐related complications, such as polymer‐induced vessel wall inflammation, inadequate or excessive endothelialization, and incomplete stent expansion or apposition [2]. In this context, drug‐coated balloons (DCB) have been introduced as an alternative treatment in various settings for PCI. The AGENT DCB (Boston Scientific) was recently approved by the US Food and Drug Administration in March 2024, making it the first coronary DCB approved in the US for treating coronary in‐stent restenosis (ISR) [3]. Nevertheless, there is still a lack of evidence regarding the optimal strategy for PCI in different clinical settings, and the long‐term outcomes of PCI with DCB versus DES remain unclear. The primary objective of this study was to perform a systematic review and meta‐analysis of randomized controlled trials (RCTs) comparing DCB and DES in patients with ISR and de‐novo lesions, including ST‐elevation myocardial infarction (STEMI) and small vessel disease (SVD), to investigate outcomes before and after 1‐year follow‐ups.

### 1.1 Methods

This study followed the Preferred Reporting Items for Systematic Reviews and Meta‐Analysis (PRISMA) standards and was registered in advance with the PROSPERO registry (CRD42024582956). Since this study is based on a secondary literature review of published RCTs, it does not require additional ethical approval.

### 1.2 Search Strategy

We systematically searched PubMed, Scopus, Embase, and the Cochrane Library databases from inception until August 2024 to identify relevant RCTs. The search was conducted using the following key terms: (“DCB” OR “DES” OR “plain old balloon angioplasty [POBA]”) AND (“CAD” OR “STEMI” OR “ISR” OR “SVD”), along with their equivalent terms, without restriction on language. More details about search queries for each database are available in the Supporting Material. Additionally, we reviewed the reference lists of included studies to ensure we identified all potentially relevant studies.

### 1.3 Data Extraction and Eligibility Criteria

In the context of ISR, we included RCTs comparing DCB and DES. For de novo lesions, studies directly comparing DCB vs. DES in STEMI and SVD were included. Studies were included if they met the following criteria: (1) RCT or post‐hoc analyses of RCTs, (2) reported at least one desired outcome comparing DCB and DES in ISR and DCB vs. DES in STEMI and SVD patients undergoing PCIs. We excluded observational studies, review articles, and non‐English studies. Additionally, studies involving the implantation of a BMS alongside a DCB in the same arm were excluded.

Four authors (F.O.S., S.H., S.S.M., and M.A.P.) independently screened the study titles and abstracts against the prespecified eligibility criteria. Any disagreements were resolved by mutual consensus with a fifth author (A.S.M.). The following variables were extracted from included studies using a pre‐determined data extraction form: study characteristics (publication year, country, center), patient characteristics (age, sex, history of diabetes, hypertension, dyslipidemia, smoking, and left ventricular ejection fraction [LVEF]), device type, device generation, and post‐procedural mean and standard deviation of angiographic late lumen loss (LLL). Binary outcomes were extracted as the number of events (target lesion revascularization [TLR], all‐cause mortality, cardiovascular mortality, myocardial infarction (MI), and device thrombosis) at 1‐year and the longest available follow‐up for >1‐year outcomes.

### 1.4 Outcomes

The primary outcome was TLR. Most trials defined TLR as any ischemia‐driven revascularization procedure, either by PCI or coronary artery bypass grafting, involving the target lesion; in two trials [4, 5], only target vessel revascularization was assessed. The definition of revascularization in each trial is available in Supplemental Table 1. The secondary outcomes were all‐cause mortality, cardiovascular mortality, MI, device thrombosis, and LLL. All outcomes except for 6‐ to 9‐month angiographic LLL were assessed based on 1‐year and > 1‐year follow‐up intervals for patients with ISR and SVD. However, we were only able to report 1‐year outcomes for STEMI patients due to the limited number of studies with > 1‐year follow‐up.

### 1.5 Risk of Bias and Quality of Evidence

The Cochrane Collaboration's Risk of Bias‐2 (RoB‐2) tool was utilized to evaluate the risk of bias arising from each trial [6]. Two authors (R.E. and M.N.) independently evaluated the quality of each study according to established criteria. Any assessment discrepancies were resolved through consultation with a third author (A.S.M.). Furthermore, we evaluated the quality of the evidence for each outcome using the Grading of Recommendations, Assessment, Development, and Evaluations framework [7].

### 1.6 Statistical Analysis

We synthesized the data by calculating effect sizes, using odds ratios (OR) for binary outcomes and standardized mean differences (SMD) for continuous outcomes. Heterogeneity was evaluated through Higgins and Thompson's *I*² statistic, where an *I*² value > 50% indicated substantial heterogeneity. Additionally, the between‐study variance (τ²) was estimated using random‐effects models, employing the Sidik–Jonkman method for τ² estimation [8]. Furthermore, to evaluate outcomes of DCB versus DES in patients with ISR, STEMI, and SVD, we employed binary random‐effects models that applied the DerSimonian and Laird method to pool log odds ratios. Sensitivity analysis was performed using the leave‐one‐out method. According to the DCB ARC consensus [9], SVD is defined as a reference vessel diameter of less than 2.75 mm. Accordingly, we performed an additional sensitivity analysis excluding two studies that did not adopt this definition.

We conducted meta‐regression analyses to explore the influence of age, sex, history of diabetes, hypertension, dyslipidemia, smoking, and LVEF on the risk of each outcome when comparing DCB and DES interventions using a mixed‐effects model. Potential publication bias was examined visually using comparison‐adjusted funnel plots and statistically using Egger's test. All analyses were performed using the *meta*, *metabin*, *metafor*, and *ggplot2* packages in R version 4.2.1 (The R Foundation, Vienna, Austria). Statistical significance was determined at a two‐sided *p*‐value threshold of <0.05.

## 2 Results

**FIGURE 1:**
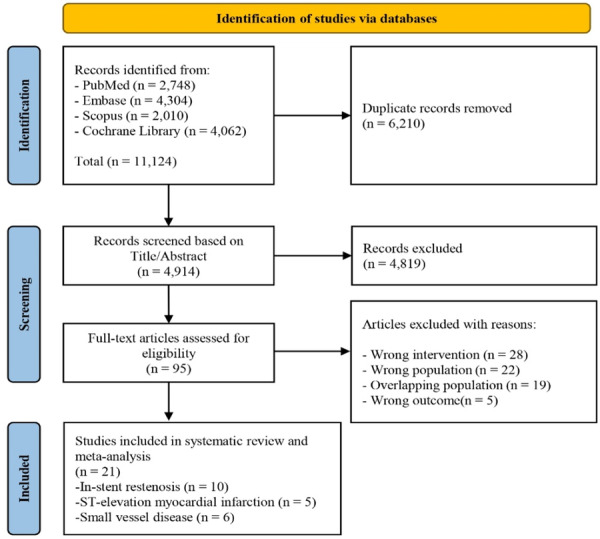
Prisma flow diagram of the study.

Our systematic search initially identified 11,124 relevant studies. After removing duplicates, 4914 studies underwent title and abstract screening. Eventually, after full‐text screening, 21 RCTs were included in our final analysis [2, 4, 5, 8, 9, 10, 11, 12, 13, 14, 15, 16, 17, 18, 19, 20, 21, 22, 23, 24, 25, 26] (Figure 1). Included studies compromised 21 RCTs with a total number of 4244 patients undergoing PCI (10 studies in the ISR arm [2, 4, 5, 10, 11, 12, 18, 22, 25, 26], five studies in the STEMI arm [8, 14, 15, 22, 23], and six studies in the SVD arm [13, 14, 17, 19, 20, 21]). These RCTs were predominantly conducted in Europe (15 studies), China (5 studies), and South Korea (1 study). Details about study and baseline characteristics, post‐procedural outcomes, and the type and generation of devices in each trial are reported in Table 1 and Supplemental Tables 2–4. Furthermore, the risk of bias assessment results of included studies and the quality of evidence for each outcome are available in Supporting Information Figure 1 and Supporting Information Table 5.

**TABLE 1:** Characteristics of the included studies.

Trial name	Year	Country	Patients	Total patients	Group	*N*, Group	Follow‐up (months/years)
ISAR‐DESIRE 3	2013	Germany	ISR (DES ISR)	402	DCB	137	10 years
					DES	131	
					POBA	134	
RIBS IV	2018	Spain	ISR (DES ISR)	309	DCB	154	3 years
					DES	155	
RIBIS V	2014	Spain	ISR (BMS ISR)	189	DCB	95	3 years
					DES	94	
TIS	2016	Czech Republic	ISR (BMS ISR)	136	DCB	68	3 years
					DES	68	
PEPCAD	2009	Germany	ISR (BMS ISR)	131	DCB	66	3 years
					DES	65	
PEPCAD China	2014	China	ISR (DES ISR)	215	DCB	109	2 years
					DES	106	
BIOLUX	2018	Germany	ISR (DES + BMS)	229	DCB	157	18 months
					DES	72	
DARE	2018	Netherlands	ISR (DES + BMS)	278	DCB	137	1 year
					DES	141	
RESTORE ISR	2019	South Korea	ISR (DES ISR)	172	DCB	86	1 year
					DES	86	
SEDUCE	2014	Belgium	ISR (BMS ISR)	50	DCB	25	1 year
					DES	25	
REVELATION trial	2019	Netherlands	STEMI	120	DCB	60	5 years
					DES	60	
Hao et al.	2021	China	STEMI	80	DCB	38	1 year
					DES	42	
Wang et al.	2022	China	STEMI	184	DCB	92	1 year
					DES	92	
Gobic et al.	2017	Croatia	STEMI	78	DCB	41	6 months
					DES	37	
DEB‐AMI	2012	Netherlands	STEMI	99	DCB	50	6 months
					DES	49	
DISSOLVE SVD	2024	China	SVD	247	DCB	129	1 year
					DES	118	
RESTORE SVD	2020	China	SVD	230	DCB	116	2 years
					DES	114	
PICCOLETO II	2023	Europe	SVD	232	DCB	118	3 years
					DES	114	
PICCOLETO	2010	Italy	SVD	57	DCB	28	9 months
					DES	29	
BASKET‐SMALL 2	2020	Europe and Australia	SVD	758	DCB	382	3 years
					DES	376	
BELLO	2012	Italy	SVD	182	DCB	90	2 years
					DES	92	

Abbreviations: BMS, bare metal stent; DCB, drug‐coated balloon; DES, drug‐eluting stent; ISR, in‐stent restenosis; POBA, plain old balloon angioplasty; STEMI, ST‐segment elevation myocardial infarction; SVD, small vessel disease.

### 2.1 ISR

**FIGURE 2:**
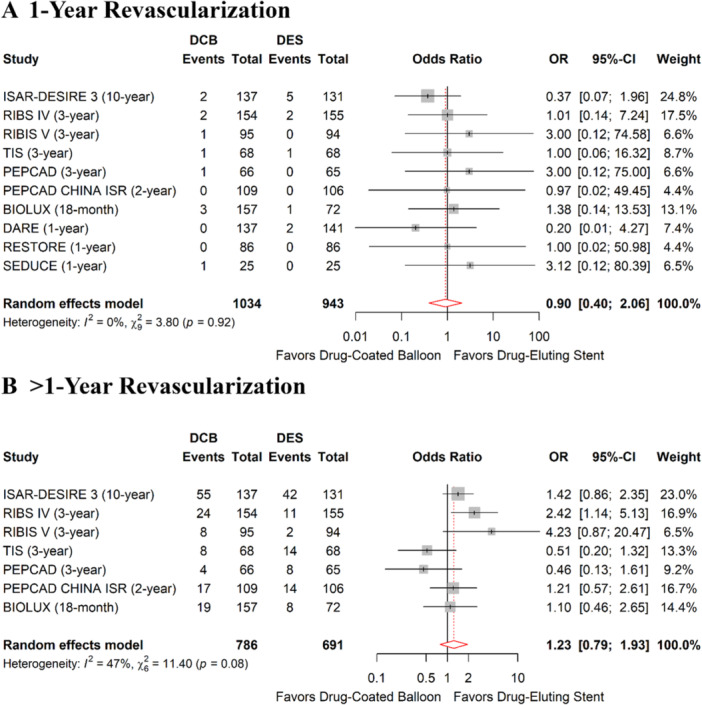
Forest plots representing meta‐analysis target lesion revascularization (TLR) between drug‐coated balloon and drug‐eluting stent in patients with in‐stent restenosis (ISR) (A) 1‐year TLR (B) > 1‐year TLR.

Our meta‐analysis included 10 RCTs involving 1,977 patients (1034 in the DCB arm, 943 in the DES arm) with ISR [2, 4, 5, 10, 11, 12, 18, 22, 25, 26]. A Paclitaxel‐coated balloon was used in all DCB arms. Time trend analysis revealed that DCB and DES interventions were associated with a similar risk of 1‐year TLR (OR: 1.36, 95% confidence interval [CI]: 0.86‐2.14, *P*: 0.19, *I²*:43.3%) and > 1‐year TLR (OR: 1.23, 95% CI: 0.79–1.93, *P*: 0.35, *I²*:47.4%) (Figure 2).

In terms of secondary outcomes, the risks of all‐cause mortality, cardiovascular mortality, MI, and stent thrombosis were comparable between DCB and DES in 1‐year and beyond 1‐year follow‐ups (Supplemental Figures 2–5). At 6–9 months angiographic follow‐up, DCB was associated with non‐significantly lower LLL compared to DES (standardized mean difference [SMD] −0.12 mm; 95% CI −0.29, 0.05; *P:* 0.17; *I* ^*2*^: 71.7%) (Supporting Information Figure 6).

Sensitivity analysis using the leave‐one‐out method indicated that excluding the TIS trial [5] would change the pooled odds ratio to a significantly higher risk of 1‐year TLR after DCB compared to DES (OR: 1.55, 95% CI: 1.02–2.34, *P*: 0.04, *I²*: 25.5%). Otherwise, the removal of no single study changed the results for other outcomes (Supporting Information Table 6).

### 2.2 STEMI

**FIGURE 3:**
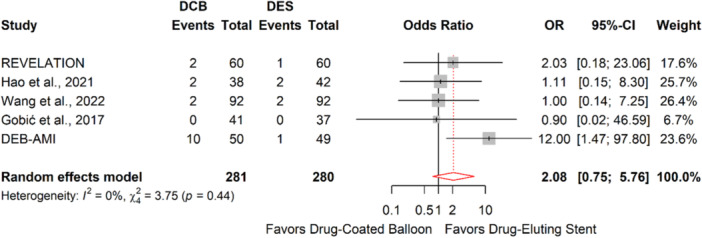
Forest plot representing meta‐analysis of 1‐year target lesion revascularization (TLR) between drug‐coated balloon and drug‐eluting stent in patients with ST‐elevation myocardial infarction (STEMI).

Our meta‐analysis included 5 RCTs involving 561 patients (281 in the DCB arm, 280 in the DES arm) with STEMI [8, 15, 16, 23, 24]. A Paclitaxel‐coated balloon was used in all DCB arms. The risk of 1‐year TLR was comparable between DCB and DES (OR: 2.08, 95% CI: 0.75–5.76, *P:* 0.16, *I²*: 0%, Figure 3). The risks of all‐cause mortality, cardiovascular mortality, MI, thrombosis, and LLL were similar between DCB and DES interventions (Supporting Information Figures 7–11). Additionally, sensitivity analysis revealed that the removal of no single study changed the results significantly (Supporting Information Table 7).

### 2.3 SVD

**FIGURE 4:**
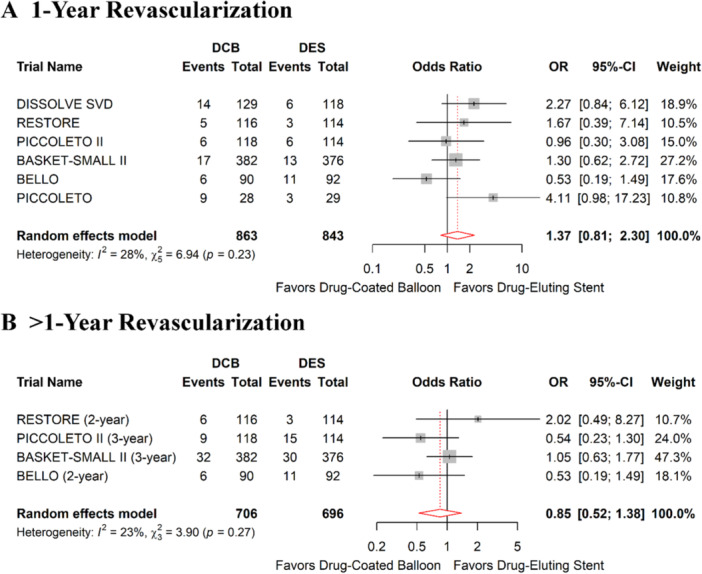
Forest plots representing meta‐analysis target lesion revascularization (TLR) between drug‐coated balloon and drug‐eluting stent in patients with small vessel disease (SVD) (A) 1‐year TLR (B) > 1‐year TLR.

Data for SVD were reported in 6 RCTs involving 1,706 patients (863 in the DCB arm, 843 in the DES arm) [13, 14, 17, 19, 20, 21]. A paclitaxel‐coated balloon was utilized in all trials. [37] The risk of 1‐year TLR was similar between DCB and DES interventions (OR: 1.37, 95% CI: 0.81–2.30, *P*: 0.23, *I²*: 27.9%, Figure 4). Sensitivity analysis using the leave‐one‐out method indicated that excluding the BELLO trial [19] would change the pooled odds ratio to a significantly higher risk of 1‐year TLR after DCB compared to DES (OR: 1.62, 95% CI: 1.02–2.60, *P*: 0.04, *I*² :0%; Supporting Information Table 8). Similar to 1‐year results, the risk of > 1‐year TLR was comparable between DCB and DES (OR: 0.85, 95% CI: 0.52–1.38, *P:* 0.51, *I²*: 23.1%, Figure 4). Moreover, excluding studies that did not adopt the DCB ARC definition for SVD, DCB became associated with a higher risk of target lesion revascularization compared with DES at 1 year (Supporting Figure 17).

Regarding secondary outcomes, DCB was associated with a lower LLL than DES (SMD: −0.38, 95% CI: −0.53 to −0.22, *P*: < 0.001, *I²*: 94.3%; Supporting Information Figure 12). The risks of all‐cause mortality, cardiovascular mortality, MI, and thrombosis did not differ between DCB and DES strategies at one‐ year or > 1‐year (Supporting Information Figures 13–16). Moreover, the sensitivity analysis showed no significant changes when individual studies were excluded for each secondary outcome (Supporting Information Table 8).

### 2.4 Meta‐Regression

Meta‐regression in patients with ISR indicated that the risk of 1‐year TLR after DCB compared to DES was increased with higher prevalence of diabetes (meta‐regression estimate ($\hat{\beta })$: 0.07; P: 0.003; *I* ^2^: 0%). In addition, male sex was associated with a higher odds of > 1‐year TLR after DCB vs. DES ($\hat{\beta }$: 0.06; *P*: 0.02; *I* ^2^: 0%) and also an increased risk of LLL after DCB versus DES ($\hat{\beta }$: 0.034; *P*: 0.001; *I* ^2^: 41.8%; Supplemental Table 9). In patients with STEMI and SVD, meta‐regression analysis showed that the modifying effect of age, gender, LVEF, history of hypertension, diabetes, dyslipidemia, and current smoking status on the risk of target outcomes was small and of uncertain magnitude (Supplemental Tables 10–12).

### 2.5 Publication Bias

No significant publication bias was observed in the funnel plot analyses for all outcomes at both the 1‐year and >1‐year follow‐ups in patients with ISR and de‐novo lesions. The corresponding funnel plots for all outcomes are shown in Supporting Information Figures 18–35.

## 3 Discussion

**FIGURE 5:**
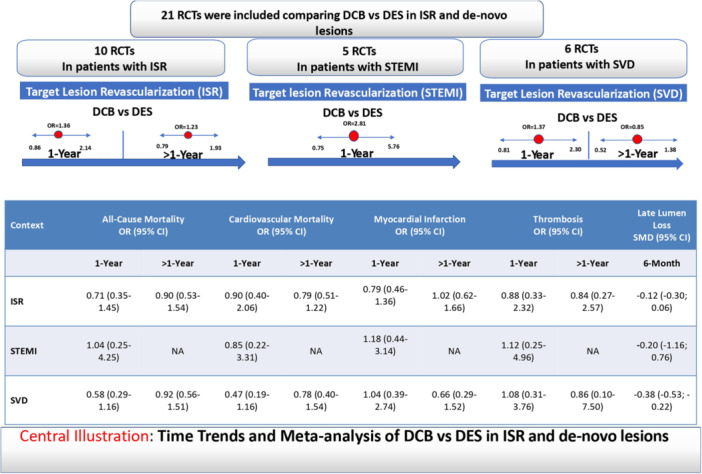
Summary and time trends of the primary and secondary outcomes in percutaneous coronary interventions. CI, confidence interval; DCB, drug‐coated balloon; DES, drug‐eluting stent; ISR, in‐stent restenosis; NA, not assessed; OR, odds ratio; RCT, randomized controlled trial; SMD, standardized mean difference; STEMI, ST‐elevation myocardial infarction; SVD, small vessel disease.

The current study presents the most comprehensive and up‐to‐date meta‐analysis of 21 RCTs comparing the widely employed strategies in patients with ISR and de novo lesions (STEMI and SVD). Time trend analysis of the ISR group indicated that both DCB and DES were associated with a similar risk of TLR at 1 year and beyond. Furthermore, DCB and DES demonstrated comparable outcomes across secondary endpoints. The meta‐analysis in patients with STEMI and SVD revealed that DCB and DES interventions were associated with similar results across primary and secondary endpoints, except for lower 6‐ to 9‐month LLL after DCB than DES in patients with SVD. Meta‐regression analysis showed that male sex and diabetes were associated with a higher risk of 1‐year TLR in DCB compared with DES in patients with ISR. (Figure 5).

### 3.1 ISR

According to our results, DCB and DES were associated with a similar risk of TLR in patients with ISR. Our results regarding > 1‐year TLR must be interpreted with extra caution. Trials reporting > 1‐year TLR had heterogenous follow‐up durations, and most of them reported 2–3‐year outcomes, while only one trial (ISAR‐DESIRE 3) [2] reported 10‐year outcomes. The long‐term benefit of these devices may diminish due to factors like the waning drug effect, delayed healing, or mechanical issues [27]. Our findings align with the DAEDALUS individual patient data meta‐analysis, which compared DCB and DES in ISR over 3 years [28]. In the primary integrated analysis of all patient‐level data, DAEDALUS reported a slightly higher risk of TLR with DCB versus DES (HR: 1.32, 95% CI: 1.02–1.70, *P*: 0.035). However, this significant association was not found in their subsequent conventional meta‐analysis of aggregated trial results, likely due to a modest effect size and moderate heterogeneity across studies [28]. This suggests the initial finding may reflect analytical sensitivity rather than a robust clinical difference. In our study, by examining 1‐year and > 1‐year trends, we found no significant difference in TLR between DCB and DES at either timeframe, further supporting the overall clinical equivalence of these strategies over time. Consistent with the DAEDALUS study, the risk of additional secondary outcomes was similar between DCB and DES.

It is also important to distinguish between ISR following DESs and that occurring after bare‐metal stents, as these two entities differ in pathophysiology and treatment response. Although our meta‐analysis did not assess outcomes according to the underlying stent type, the DAEDALUS study highlighted this distinction, showing that the relative efficacy of DCB versus DES varied between these subgroups. This observation emphasizes the need for future investigations to separately evaluate DES‐ISR and BMS‐ISR, as differences in vessel healing, neointimal hyperplasia, and polymer‐related inflammation may influence the comparative performance of these devices [28].

### 3.2 STEMI

Our study confirms that no differences exist between DCB and DES, as observed in previous meta‐analyses [29], but uniquely focuses on STEMI patients. DCB offers potential advantages in acute settings, such as better endothelial healing, less inflammation, and reduced risk of under‐sizing vessels or slow flow phenomena [24]. Additionally, DCB may allow for shorter dual antiplatelet therapy, benefiting high‐risk bleeding patients [30]. However, DCB use may increase the risk of distal embolization and no‐reflow, necessitating proper lesion preparation and operator expertise to minimize complications [16, 23, 24]. Future trials are needed to further evaluate DCB in STEMI patients with complex lesions.

### 3.3 SVD

Performing PCI with DES in small‐caliber vessels is associated with higher rates of ISR, late LLL, and repeat revascularization [20]. The absence of a permanent stent structure with DCB reduces the risk of chronic vessel injury and inflammation, which may lead to better long‐term outcomes, as shown in the BELLO [19] and PICCOLETO 2 [14] trials. Our findings align with recent meta‐analyses and the subgroup analysis from the REC‐CAGEFREE trial, showing comparable outcomes between DCB and DES in SVD [31, 32]. Our findings in SVD are consistent with the ANDROMEDA [31] individual patient data meta‐analysis, which also showed similar rates of TLR between DCB and DES. While ANDROMEDA reported fewer major adverse cardiovascular events with DCB, we did not assess MACE due to heterogeneous definitions across trials. Nevertheless, the comparable outcomes observed in our analysis reinforce the role of DCB as a safe and effective alternative to DES in small vessel interventions.

### 3.4 Study Limitations

This meta‐analysis, while comprehensive, is subject to certain limitations. First, despite minimal overall heterogeneity, there were methodological differences among the included trials, such as variations in follow‐up duration, entry criteria, and primary endpoints, which may affect the generalizability of the findings. Second, the small number of trials with > 1‐year follow‐up in ISR and SVD restricted our ability to fully understand the long‐term outcomes, necessitating further studies with extended follow‐ups to better assess the durability of these treatments. Third, due to the limited data availability, we could not include > 1‐year outcomes for patients with STEMI, which limits our analysis of the long‐term efficacy of DCB and DES in this specific population. Fourth, our analysis did not account for the type of polymer, excipient, or drug in DES or the differences across DES generations, which could play a significant role in the observed outcomes. Fifth, focusing exclusively on randomized trials while minimizing bias restricts the generalizability of our results to broader patient populations excluded from these studies. Furthermore, we could not include the SVD subgroup analysis of the REC‐CAGEFREE trial [31] published in 2024 because they solely applied their subgroup analysis to their primary outcome of composite endpoints. These limitations underscore the need for further research with more diverse populations and longer follow‐up periods to confirm and extend our findings.

## 4 Conclusions

In conclusion, our meta‐analysis demonstrated that DCB and DES were associated with a similar risk of TLR in patients with ISR and SVD at 1‐year and beyond. Further, DCB and DES demonstrated a comparable risk of TLR in patients with STEMI at 1‐year. Notably, no differences were observed in the risk of all‐cause mortality, cardiovascular mortality, MI, and thrombosis between DCB and DES.

## Author Contributions

Reza Hosseini Dolamaa and Kaveh Hosseini are responsible for conceptualization. Sina Kazemian, Hamidreza Soleimani, Masih Tajdini, Behnam Tehrani, Dhaval Kolte, Islam Elgendy, Sandeep Basavarajaiah, Azeem Latib, Yasar Sattar, M. Chadi Alraies, and Kaveh Hosseini contributed to the methodology. Rasoul Ebrahimi and Mohammad Sadeq Najafi contributed to the Software. Saman Soltani Moghadam, Rasoul Ebrahimi, Fatemeh Ojaghi Shirmard, and Mohammad Sadeq Najafi contributed to data curation. Arman Soltani Moghadam, Reza Hosseini Dolama, and Kaveh Hosseini are responsible for the investigation. Shayan Shojaei, Saman Soltani Moghadam, Reza Hosseini Dolama, Sayed Morteza Ali Pourfaraji, Mohammad Sadeq Najafi, and Kaveh Hosseini contributed to the validation. Arman Soltani Moghadam and Sina Kazemian contributed to formal analysis. Hamidreza Soleimani, Masih Tajdini, Behnam Tehrani, Dhaval Kolte, Islam Elgendy, Sandeep Basavarajaiah, Azeem Latib, Yasar Sattar, M. Chadi Alraies, and Kaveh Hosseini contributed to supervision. Shayan Shojaei, Saman Soltani Moghadam, Reza Hosseini Dolama, Fatemeh Ojaghi Shirmard, Sayed Morteza Ali Pourfaraji, and Sara Hobaby are responsible for visualization. Saman Soltani Moghadam, Sayed Morteza Ali Pourfaraji, Sara Hobaby, and Kaveh Hosseini contributed to project administration. Rasoul Ebrahimi, Fatemeh Ojaghi Shirmard, Sara Hobaby, and Kaveh Hosseini contributed to resources. Arman Soltani Moghadam and Shayan Shojaei contributed to writing – original draft. Arman Soltani Moghadam, Sina Kazemian, Hamidreza Soleimani, Masih Tajdini, Behnam Tehrani, Dhaval Kolte, Islam Elgendy, Sandeep Basavarajaiah, Azeem Latib, Yasar Sattar, M. Chadi Alraies, and Kaveh Hosseini contributed to writing – review and editing. We confirm that all authors have read and approved the final version of the manuscript, and the corresponding author (Kaveh Hosseini, MD, MPH) has full access to all of the data in this study and takes complete responsibility for the integrity of the data and the accuracy of the data analysis.

## Conflicts of Interest

The authors declare no conflicts of interest.

## Transparency Statement

The lead author, Kaveh Hosseini, affirms that this manuscript is an honest, accurate, and transparent account of the study being reported; that no important aspects of the study have been omitted; and that any discrepancies from the study as planned (and, if relevant, registered) have been explained.

## Supporting information

Supporting File

## Data Availability

The data supporting this study's findings are available from the corresponding author upon reasonable request.
