# Supplementary material for: Outcomes of Drug‐Coated Balloon Versus Drug‐Eluting Stent for In‐Stent Restenosis and De‐Novo Lesions: A Meta‐Analysis of Randomized Controlled Trials
Source: Health Sci Rep. 2026 Apr 24;9(5):e72061. doi: 10.1002/hsr2.72061 (PMC13109081; doi:10.1002/hsr2.72061)
Supplement: Supplementary file 1 — Supporting File [file HSR2-9-e72061-s001.docx]

**Outcomes of Drug-Coated Balloon Versus Drug-Eluting Stent for In-Stent Restenosis and De-Novo Lesions: A Meta-Analysis of Randomized Controlled Trials**

Contents

[Search Strategy 2](#_Toc195350588)

[PubMed (August 2024) 2](#_Toc195350589)

[Embase (August 2024) 2](#_Toc195350590)

[Scopus (August 2024) 3](#_Toc195350591)

[Cochrane (August 2024) 3](#_Toc195350592)

[Total Results 3](#_Toc195350593)

[Definition of Outcome 4](#_Toc195350594)

[Supplemental Table 1. 4](#_Toc195350595)

[Characteristics of the Included Studies 5](#_Toc195350596)

[Supplemental Table 2. 5](#_Toc195350597)

[Supplemental Table 3. 7](#_Toc195350598)

[Supplemental Table 4 9](#_Toc195350599)

[Quality Assessment 10](#_Toc195350600)

[Supplemental Figure 1. 10](#_Toc195350601)

[Supplemental Table 5. 11](#_Toc195350602)

[Main Results of In-Stent Restenosis (ISR) 15](#_Toc195350604)

[Main Results of ST-Elevation Myocardial Infarction (STEMI) 20](#_Toc195350605)

[Main Results of Small Vessel Disease (SVD) 22](#_Toc195350606)

[Sensitivity Analysis 26](#_Toc195350607)

[Sensitivity Analysis in ISR Patients 26](#_Toc195350608)

[Sensitivity Analysis in STEMI Patients 27](#_Toc195350609)

[Sensitivity Analysis in SVD Patients 29](#_Toc195350610)

[Meta-Regression Analysis 31](#_Toc195350611)

[Publication Bias 34](#_Toc195350612)

[ISR 34](#_Toc195350613)

[STEMI 37](#_Toc195350614)

[SVD 40](#_Toc195350615)

# Search Strategy

## PubMed (August 2024)

| # | **Syntax** | **Results** |
| --- | --- | --- |
| #1 | "polymer-free paclitaxel-eluting stent"[Title/Abstract] OR "polymer-free DES"[Title/Abstract] OR "SeQuent Please DCB"[Title/Abstract] OR "Everolimus eluting stent"[Title/Abstract] OR "Sirolimus eluting stent"[Title/Abstract] OR "Paclitaxel eluting stent"[Title/Abstract] OR "Drug eluting stent"[Title/Abstract] OR "Drug eluting balloon"[Title/Abstract] OR "Drug coated balloon"[Title/Abstract] OR "Paclitaxel eluting balloon"[Title/Abstract] OR "Paclitaxel coated balloon"[Title/Abstract] OR "Uncoated balloon"[Title/Abstract] OR "Uncoated balloon angioplasty"[Title/Abstract] OR "Balloon angioplasty"[Title/Abstract] OR "drug eluting stents"[MeSH Terms] OR "angioplasty, balloon, coronary"[MeSH Terms] OR “plain old balloon angioplasty” [Title/Abstract] | 56,644 |
| #2 | "ST segment elevation myocardial infarction"[Title/Abstract] OR "ST elevation myocardial infarction"[Title/Abstract] OR "Acute myocardial infarction"[Title/Abstract] OR "Acute myocardial injury"[Title/Abstract] OR "Acute myocardial ischemia"[Title/Abstract] OR "ST elevation myocardial infarction"[MeSH Terms] OR "myocardial infarction"[MeSH Terms] OR "coronary restenosis"[Title/Abstract] OR "coronary artery restenosis"[Title/Abstract] OR "in-stent restenosis"[Title/Abstract] OR "coronary restenosis"[MeSH Terms] | 241,758 |
| #3 | "randomized controlled study"[Title/Abstract] OR "randomized controlled trial"[Title/Abstract] OR "RCT"[Title/Abstract] OR "randomized controlled trial"[Publication Type] | 691,495 |
| #4 | #1 AND #2 AND #3 | **2,748** |

## Embase (August 2024)

| **#** | **Syntax** | **Results** |
| --- | --- | --- |
| #1 | 'drug-coated balloon'/exp OR 'drug coated balloon':ti,ab OR 'balloon angioplasty':ti,ab OR 'drug coated balloon angioplasty'/exp OR 'drug-coated catheter':ti,ab OR 'drug eluting stent'/exp OR 'coronary drug-eluting stents':ti,ab OR dcb:ti,ab OR 'sirolimus eluting coronary stent'/exp OR 'sirolimus eluting stents':ti,ab OR 'uncoated balloon':ti,ab OR 'uncoated balloon angioplasty':ti,ab OR 'angioplasty uncoated balloon':ti,ab OR 'sirolimus coated balloon'/exp OR 'paclitaxel eluting coronary stent'/exp OR 'paclitaxel coated catheter':ti,ab OR 'paclitaxel coated balloon':ti,ab OR 'plain balloon angioplasty'/exp OR 'plain balloon':ti,ab OR 'plain balloons':ti,ab OR 'everolimus eluting coronary stent'/exp OR des:ti,ab | 117,378 |
| #2 | 'in-stent restenosis'/exp OR 'stent restenosis'/exp OR isr:ti,ab OR 'st segment elevation myocardial infarction'/exp OR 'coronary restenosis'/exp OR 'coronary artery restenosis':ti,ab OR 'in stent restenosis' OR 'acute heart infarction'/exp OR 'st elevation mi':ti,ab OR stemi:ti,ab OR 'acute myocardial infarction':ti,ab OR 'stent restenosis':ti,ab OR 'in stent stenosis'/exp OR ‘small vessel disease’/exp OR ‘small coronary vessel’/exp | 212,365 |
| #3 | 'randomized controlled trial'/exp OR rct: ti, ab OR 'randomized clinical trial’: ti, ab OR 'randomized controlled study’: ti, ab | 1,136,972 |
| #4 | #1 AND #2 AND # 3 | **2,304** |

## Scopus (August 2024)

| **#** | **Syntax** | **Results** |
| --- | --- | --- |
| #1 | ( TITLE-ABS-KEY ( "Everolimus eluting stent" ) OR TITLE-ABS-KEY ( "Sirolimus eluting stent" ) OR TITLE-ABS-KEY ( "Paclitaxel eluting stent" ) OR TITLE-ABS-KEY ( "Drug eluting stent" ) OR TITLE-ABS-KEY ( "Drug eluting balloon" ) OR TITLE-ABS-KEY ( "Drug coated balloon" ) OR TITLE-ABS-KEY ( "Paclitaxel eluting balloon" ) OR TITLE-ABS-KEY ( "Paclitaxel coated balloon" ) OR TITLE-ABS-KEY ( "Uncoated balloon" ) OR TITLE-ABS-KEY ( "Uncoated balloon angioplasty" ) OR TITLE-ABS-KEY ( "Balloon angioplasty" ) ) | 40,169 |
| #2 | ( TITLE-ABS-KEY ( "ST segment elevation myocardial infarction" ) OR TITLE-ABS-KEY ( "STEMI" ) OR TITLE-ABS-KEY ( "ST segment elevation heart infarction" ) OR TITLE-ABS-KEY ( "ST elevation myocardial infarction" ) OR TITLE-ABS-KEY ( "Acute myocardial infarction" ) OR TITLE-ABS-KEY ( "Acute myocardial injury" ) OR TITLE-ABS-KEY ( "Acute myocardial ischemia" ) OR TITLE-ABS-KEY ( "coronary restenosis" ) OR TITLE-ABS-KEY ( "in-estent coronary restenosis" ) OR TITLE-ABS-KEY ( "in-estent restenosis" ) OR TITLE-ABS-KEY ( "coronary artery restenosis" ) OR TITLE-ABS-KEY (“small vessel disease” ) OR TITLE-ABS-KEY (“small coronary vessel”)) | [142,950](https://www-scopus-com.ezproxy.udes.edu.co/search/history/results.uri?origin=searchhistory&shid=7) |
| #3 | (TITLE-ABS-KEY ( "randomized controlled trial" ) OR TITLE-ABS-KEY ( "RCT" ) OR TITLE-ABS-KEY ( "randomized controlled study" ) ) | 1,043,517 |
| #4 | #1 AND #2 AND # 3 | **2,010** |

## Cochrane (August 2024)

| **#** | **Syntax** | **Results** |
| --- | --- | --- |
| #1 | MeSH descriptor: [Drug-Eluting Stents] explode all trees OR ("Drug-Eluting Stent"): ti,ab,kw OR ("Balloon Angioplasty"): ti,ab,kw OR ("uncoated balloon"): ti,ab,kw OR ("drug coated balloon"): ti,ab,kw OR ("plain old balloon angioplasty"): ti,ab,kw | 11147 |
| #2 | MeSH descriptor: [ST Elevation Myocardial Infarction] explode all trees OR ("ST Elevation Myocardial Infarction"): ti,ab,kw OR ("Coronary Restenosis"): ti,ab,kw OR ("in-stent restenosis"): ti,ab,kw OR ("small vessel disease"): ti,ab,kw OR ("small coronary vessels"): ti,ab,kw | 26942 |
| #3 | MeSH descriptor: [Randomized Controlled Trial] explode all trees OR ("Randomized Controlled Trial"): ti,ab,kw OR ("RCT"): ti,ab,kw | 1022278 |
| #4 | #1 AND #2 AND # 3 | **4062** |

## Total Results

| **Database** | **Results (No.)** |
| --- | --- |
| PubMed | 2748 |
| Embase | 2304 |
| Scopus | 2010 |
| The Cochrane Library | 4062 |
| **Total** | **11124** |

# Definition of Outcome

## Supplemental Table 1. Definition of revascularization in each trial

| **Study** | **Group** | **Outcome** | **Definition of outcome** |
| --- | --- | --- | --- |
| ISAR-DESIRE 3 | ISR | TLR | Any ischemia-driven revascularization procedure, either by PCI or CABG, involving the target lesion |
| RIBS IV | ISR | TLR | Any ischemia-driven revascularization procedure, either by PCI or CABG, involving the target lesion |
| RIBIS V | ISR | TLR | Any ischemia-driven revascularization procedure, either by PCI or CABG, involving the target lesion |
| TIS | ISR | TVR | The specified definition was not reported |
| PEPCAD | ISR | TLR | Any ischemia-driven revascularization procedure, either by PCI or CABG, involving the target lesion |
| PEPCAD CHINA | ISR | TLR | The specified definition was not reported |
| BIOLUX | ISR | TLR | Any ischemia-driven revascularization procedure, either by PCI or CABG, involving the target lesion |
| DARE | ISR | TVR | Any ischemia-driven revascularization procedure, either by PCI or CABG, involving the target vessel |
| Restore ISR | ISR | TLR | Any ischemia-driven revascularization procedure, either by PCI or CABG, involving the target lesion |
| SEDUCE | ISR | TLR | Any ischemia-driven revascularization procedure, either by PCI or CABG, involving the target lesion |
| REVELATION | STEMI | TLR | Any ischemia-driven revascularization procedure, either by PCI or CABG, involving the target lesion |
| Hao et al., 2021 | STEMI | TLR | The specified definition was not reported |
| Wang et al., 2022 | STEMI | TLR | The specified definition was not reported |
| Gobić et al., 2017 | STEMI | TLR | The specified definition was not reported |
| DEB-AMI | STEMI | TLR | Target lesion revascularization was defined as any repeat percutaneous or surgical intervention due to a restenosis in the treated segment (including the stent and 5mm proximal and distal). A TLR was considered clinically indicated in case of restenosis 50% by quantitative coronary angiography (QCA), associated with recurrent angina and/or objective signs of silent ischemia (stress tests or fractional flow reserve), or in case of restenosis 70% by QCA without the aforementioned signs or symptoms. |
| DISSOLVE SVD | SVD | TLR | The specified definition was not reported |
| RESTORE SVD | SVD | TLR | Any ischemia-driven revascularization procedure, either by PCI or CABG, involving the target lesion |
| PICCOLETO II | SVD | TLR | The Specified definition was not reported |
| BASKET-SMALL II | SVD | TLR | The specified definition was not reported |
| BELLO | SVD | TLR | Any ischemia-driven revascularization procedure, either by PCI or CABG, involving the target lesion |
| PICCOLETO | SVD | TLR | The Specified definition was not reported |

Abbreviations: CABG: coronary artery bypass graft; ISR: In-stent restenosis; PCI: percutaneous coronary intervention; STEMI: ST-Segment Elevation Myocardial Infarction; SVD: small vessel disease; TLR: target lesion revascularization; TLV: target vessel revascularization

# Characteristics of the Included Studies

## **Supplemental Table 2.** Baseline characteristics of patients in each trial

| **Trial Name** | **Patients** | **Group** | **N,** | **Age** | **Male** | **BMI** | **LVEF** | **N,** | **N,** | **N,** | **N,** | **N,** | **N, previous CABG** | **N, previous stroke** | **N, previous MI** | **N, peripheral artery disease** | **N, chronic renal failure** |
| --- | --- | --- | --- | --- | --- | --- | --- | --- | --- | --- | --- | --- | --- | --- | --- | --- | --- |
|  |  |  | **Group** | **(years)** |  | **(kg/m²)** |  | **Hypertension** | **Diabetes** | **Smoker** | **Dyslipidemia** | **previous PCI** |  |  |  |  |  |
|  |  |  |  | **(mean± Sd)** |  | **(mean± Sd)** |  |  |  |  |  |  |  |  |  |  |  |
| **ISAR-DESIRE 3** | ISR (DES ISR) | **DCB** | 137 | 67.7±10.4 | 105 | NR | 53.6±9.8 | 105 | 56 | 19 | 108 | NR | 15 | NR | 53 | NR | NR |
|  |  | **DES** | 131 | 68.8±10.0 | 88 | NR | 54.5±9.9 | 101 | 61 | 15 | 103 | NR | 32 | NR | 50 | NR | NR |
|  |  | **POBA** | 134 | 67.1±9.3 | 95 | NR | 53.2±9.9 | 90 | 50 | 22 | 102 | NR | 24 | NR | 57 | NR | NR |
| **RIBS IV** | ISR (DES ISR) | **DCB** | 154 | 66±10 | 127 | NR | NR | 110 | 75 | 89 | 110 | NR | NR | NR | NR | NR | NR |
|  |  | **DES** | 155 | 66±10 | 130 | NR | NR | 121 | 66 | 87 | 121 | NR | NR | NR | NR | NR | NR |
| **RIBIS V** | ISR (BMS ISR) | **DCB** | 95 | 67±11 | 82 | NR | 58±13 | 68 | 30 | 56 | 69 | NR | NR | NR | NR | NR | NR |
|  |  | **DES** | 94 | 64±12 | 82 | NR | 59±12 | 68 | 19 | 70 | 62 | NR | NR | NR | NR | NR | NR |
| **TIS** | ISR (BMS ISR) | **DCB** | 68 | 65.6±10.9 | 43 | 28.7±4.0 | 49.74±11.95 | NA | 17 | 31 | NA | NR | 3 | NR | 43 | NR | 2 |
|  |  | **DES** | 68 | 65.5±10.6 | 46 | 29.3±4.2 | 49.57±11.44 | NA | 18 | 29 | NA | NR | 6 | NR | 41 | NR | 7 |
| **PEPCAD** | ISR (BMS ISR) | **DCB** | 66 | 64.6±9.7 | 48 | NR | NR | 53 | 22 | 16 | 52 | NR | NR | NR | NR | NR | NR |
|  |  | **DES** | 65 | 65.1±8.7 | 50 | NR | NR | 54 | 17 | 15 | 46 | NR | NR | NR | NR | NR | NR |
| **PEPCAD China** | ISR (DES ISR) | **DCB** | 109 | 61.8±9.3 | 88 | NR | 61.7±8.50 | 78 | 44 | 23 | 38 | NR | 3 | NR | 53 | NR | NR |
|  |  | **DES** | 106 | 62.1±9.3 | 86 | NR | 62.3±8.60 | 69 | 35 | 27 | 35 | NR | 0 | NR | 37 | NR | NR |
| **BIOLUX** | ISR (DES + BMS) | **DCB** | 157 | 67.2±9.9 | 119 | NR | NR | 144 | 48 | 104 | 134 | NR | NR | 10 | 93 | NR | 17 |
|  |  | **DES** | 72 | 69.4±8.8 | 49 | NR | NR | 70 | 24 | 42 | 62 | NR | NR | 1 | 35 | NR | 8 |
| **DARE** | ISR (DES + BMS) | **DCB** | 137 | 66±11 | 98 | NR | NR | 87 | 42 | 23 | 84 | NR | 19 | NR | 72 | NR | 9 |
|  |  | **DES** | 141 | 65±10 | 118 | NR | NR | 94 | 46 | 18 | 81 | NR | 22 | NR | 73 | NR | 10 |
| **RESTORE ISR** | ISR (DES ISR) | **DCB** | 86 | 67±10 | 61 | NR | 59.4±8.4 | 60 | 43 | 40 | 49 | NR | NR | NR | 26 | NR | NR |
|  |  | **DES** | 86 | 66±9 | 62 | NR | 59.9±7.8 | 65 | 38 | 37 | 53 | NR | NR | NR | 22 | NR | NR |
| **SEDUCE** | ISR (BMS ISR) | **DCB** | 25 | 67.6±7.7 | 18 | 26.9 | NR | NA | 6 | 5 | 24 | NR | NR | 2 | 12 | NR | NR |
|  |  | **DES** | 25 | 64.2±11 | 25 | 27.4 | NR | NA | 1 | 3 | 24 | NR | NR | 2 | 10 | NR | NR |
| **REVELATION trial** | STEMI | **DCB** | 60 | 57.4±9.2 | 52 | 26.7±3.5 | 57.1±6.2 | 18 | 8 | 36 | 10 | 2 | 0 | NR | NR | 1 | NR |
|  |  | **DES** | 60 | 57.3±8.3 | 52 | 27.4±4.4 | 58.4±7.1 | 19 | 4 | 30 | 8 | 0 | 0 | NR | NR | 0 | NR |
| **Hao et.al** | STEMI | **DCB** | 38 | 59±11 | 30 | 26±5 | 48±11 | 11 | 15 | 11 | NA | NR | NR | NR | NR | NR | NR |
|  |  | **DES** | 42 | 56±11 | 35 | 25±12 | 45±8 | 9 | 11 | 13 | NA | NR | NR | NR | NR | NR | NR |
| **Wang et.al** | STEMI | **DCB** | 92 | 49.20±10.59 | 88 | NR | NR | 67 | 71 | 71 | 61 | NR | NR | NR | NR | NR | NR |
|  |  | **DES** | 92 | 49.60±8.82 | 84 | NR | NR | 65 | 79 | 78 | 58 | NR | NR | NR | NR | NR | NR |
| **Gobic et.al** | STEMI | **DCB** | 41 | 56.6±13.2 | 26 | NR | NR | 13 | 4 | 15 | 5 | NR | NR | NR | NR | NR | NR |
|  |  | **DES** | 37 | 54.3±10.6 | 30 | NR | NR | 13 | 2 | 21 | 7 | NR | NR | NR | NR | NR | NR |
| **DEB-AMI** | STEMI | **DCB** | 50 | 59.7±9.9 | 41 | NR | NR | 17 | 3 | 19 | 13 | 1 | 0 | NR | 1 | NR | NR |
|  |  | **DES** | 49 | 55.9±9.7 | 41 | NR | NR | 15 | 2 | 28 | 16 | 2 | 0 | NR | 2 | NR | NR |
| **DISSOLVE SVD** | SVD | **DCB** | 129 | 60.2±9.5 | 94 | 25.8±3.4 | 62.4±7.8 | 95 | 46 | 27 | 58 | NR | 1 | 10 | 33 | 8 | NR |
|  |  | **DES** | 118 | 60.1±9.3 | 82 | 25.2±3.0 | 61.7±7.8 | 89 | 45 | 25 | 64 | NR | 1 | 16 | 27 | 7 | NR |
| **RESTORE SVD China** | SVD | **DCB** | 116 | 60.1±10.5 | 77 | 25.6±3.2 | 60.6±7.3 | 78 | 46 | 34 | 61 | 45 | 0 | 8 | 26 | NR | NR |
|  |  | **DES** | 114 | 60.5±10.8 | 88 | 25.4±3.1 | 59.9±6.9 | 86 | 48 | 36 | 55 | 38 | 1 | 14 | 28 | NR | NR |
| **PICCOLETO II** | SVD | **DCB** | 118 | 64 (48-80) | 83 | NR | 58 (48-68) | 77 | 45 | 23 | 72 | 59 | 4 | NA | 45 | NR | 4 |
|  |  | **DES** | 114 | 66 (50-82) | 87 | NR | 58 (51-65) | 76 | 40 | 19 | 63 | 60 | 4 | NA | 34 | NR | 12 |
| **PICCOLETO** | SVD | **DCB** | 28 | 68±9 | 22 | NR | NR | 21 | 13 | NA | 17 | 3 | 3 | NA | 5 | NR | NR |
|  |  | **DES** | 29 | 67±10 | 22 | NR | NR | 20 | 11 | NA | 13 | 4 | 4 | NA | 6 | NR | NR |
| **BASKET-SMALL 2** | SVD | **DCB** | 382 | 67.69±10.34 | 295 | 28.42±4.5 | 60 (50–60) | 324 | 122 | 82 | 262 | 235 | 37 | 16 | 160 | 27 | 54 |
|  |  | **DES** | 376 | 67.18±10.33 | 262 | 28.1±4.5 | 60 (55–65) | 332 | 130 | 72 | 259 | 241 | 34 | 23 | 133 | 26 | 59 |
| **BELLO** | SVD | **DCB** | 90 | 64.8±8.5 | 72 | NR | NR | 72 | 39 | 15 | 71 | 52 | 9 | NA | 46 | NR | NR |
|  |  | **DES** | 92 | 66.4±9.0 | 71 | NR | NR | 75 | 35 | 10 | 73 | 39 | 12 | NA | 33 | NR | NR |

Abbreviations: BMI: Body mass index, BMS: bare metal stent, CABG: coronary artery bypass graft, DCB: Drug-coated Balloon; DES: Drug eluting stent; ISR: In-stent restenosis; LVEF: Left ventricular ejection fraction; NR: Not reported; PCI: percutaneous coronary intervention; PES: paclitaxel-eluting stent; POBA= plain old balloon angioplasty; SES: sirolimus-eluting stent; STEMI: ST-Segment Elevation Myocardial Infarction; SVD: small vessel disease.

Supplemental Table 3. Pre- and post-angiographic findings of the included studies

| **Study** | **Group** | **Total patients** | **Arms** | **LAD** | **LCX** | **RCA** | **Lesion length (mm)** | **Pre-DS %** | **Post- DS %** | **Pre -RVD (mm)** | **Post -RVD (mm)** | **Pre -MLD (mm)** | **Post- MLD (mm)** |
| --- | --- | --- | --- | --- | --- | --- | --- | --- | --- | --- | --- | --- | --- |
| **ISAR-DESIRE 3** | ISR | 402 | **DCB (137)** | 59 | 54 | 59 | NA | 64.4±16.8 % | 18.5±8.3 % | 2.75±0.50 | NA | 0.97±0.48 | 2.29±0.44 |
|  |  |  | **DES (131)** | 50 | 61 | 56 | NA | 66.7±16.5 % | 12.8±7.8 % | 2.80±0.49 | NA | 0.93±0.50 | 2.53±0.48 |
|  |  |  | **POBA (134)** | 52 | 56 | 52 | NA | 67.7±15.7 % | 23.3±12.6 % | 2.72±0.45 | NA | 0.88±0.49 | 2.10±0.49 |
| **RIBS IV** | ISR | 309 | **DCB (154)** | 77 | 27 | 43 | 10.4±5.6 | 69±17 % | 18±10 % | 2.59±0.5 | 2.58±0.5 | 0.79±0.4 | 2.10±0.4 |
|  |  |  | **DES (155)** | 71 | 34 | 45 | 10.7±5.4 | 72±15 % | 13±11 % | 2.67±0.5 | 2.55±0.5 | 0.75±0.4 | 2.22±0.50 |
| **RIBS V** | ISR | 189 | **DCB (95)** | 35 | 21 | 37 | 13.7±7 | 61±14 % | 18±11 % | 2.64±0.6 | 2.69±0.6 | 1.02±0.4 | 2.20±0.5 |
|  |  |  | **DES (94)** | 37 | 22 | 32 | 13.8±6 | 65±13 % | 9±13 % | 2.64±0.6 | 2.68±0.5 | 0.93±0.4 | 2.50±0.4 |
| **TIS** | ISR | 136 | **DCB (68)** | 35 | 16 | 22 | NA | 71.8± 13.9 % | 19.5±7.4% | 2.64±0.47 | 2.79±0.41 | 0.92±0.45 | 2.18±0.39 |
|  |  |  | **DES (68)** | 40 | 10 | 22 | NA | 78±13.4 % | 16.3±5.9% | 2.66±0.45 | 3.01±0.40 | 0.79±0.48 | 2.51±0.38 |
| **PEPCAD** | ISR | 131 | **DCB (66)** | 20 | 24 | 22 | 15.7±6.6 | 73.9±8.8% | 19.5±9.9% | 2.85±0.39 | NA | 0.74±0.27 | 2.30±0.40 |
|  |  |  | **DES (65)** | 28 | 29 | 17 | 15.4±6.6 | 72.8±9.4% | 11.2±8.1% | 2.83±0.36 | NA | 0.77±0.30 | 2.56±0.41 |
| **PEPCAD-China** | ISR | 215 | **DCB (109)** | 47 | 21 | 45 | 12.52±6.55 | 68.26±12.47% | 10.51±7.2% | 2.66±0.38 | 2.59±0.40 | 0.85±0.38 | 2.39±0.37 |
|  |  |  | **DES (106)** | 61 | 13 | 37 | 13.08±7.13 | 68.43±13.25% | 7.05±6.28% | 2.72±0.44 | 2.67±0.44 | 0.86±0.41 | 2.56±0.44 |
| **BIOLUX** | ISR | 229 | **DCB (157)** | NA | NA | NA | 5.8±4.0 | 67.2±13.5 % | 27.1±10.2% | 3.0±0.4 | NA | 1.0±0.5 | 2.2±0.4 |
|  |  |  | **DES (72)** | NA | NA | NA | 7.2±6.1 | 68.9±14.7 % | 16.3±13.5% | 2.9±0.5 | NA | 0.9±0.5 | 2.4±0.6 |
| **DARE** | ISR | 278 | **DCB (137)** | 56 | 28 | 51 | NA | 69.7±11.8 % | 26.8±12.0% | 2.56±0.43 | NA | 0.77±0.33 | 1.86±0.38 |
|  |  |  | **DES (141)** | 55 | 34 | 49 | NA | 69.3±12.5 % | 15.6±11.7% | 2.59±0.54 | NA | 0.79±0.35 | 2.2±0.41 |
| **RESTORE ISR** | ISR | 172 | **DCB (86)** | 48 | 13 | 24 | 18.1±9.7 | 77±17 % | 23±11% | 2.85±0.50 | 2.76±0.49 | 0.63±0.40 | 2.12±0.43 |
|  |  |  | **DES (86)** | 52 | 11 | 21 | 17.4±11.4 | 79±13 % | 13±10% | 3.06±0.45 | 2.95±0.46 | 0.63±0.42 | 2.54±0.41 |
| **SEDUCE** | ISR | 50 | **DCB (25)** | 6 | 5 | 13 | NA | 67.7±18.4 % | 17.4±11.9% | 3.0±0.48 | NA | 0.98±0.6 | 2.41±0.39 |
|  |  |  | **DES (25)** | 11 | 7 | 6 | NA | 79.4±13.5 % | 10.5±11.5% | 2.84±0.44 | NA | 0.57±0.37 | 2.6±0.43 |
| **REVELATION** | STEMI | 120 | **DCB (60)** | 32 | 20 | 48 | 22.6± 5.9 | 85.3±8.4 % | 28.4±6.1 % | 2.92±0.39 | 2.94±0.38 | 0.29±0.15 | 2.11±0.26 |
|  |  |  | **DES (60)** | 40 | 13 | 47 | 23.1± 6.7 | 86.7±9.2 % | 13.2±5.4 % | 2.99±0.37 | 3.01±0.36 | 0.27±0.14 | 2.85±0.32 |
| **Hao et.al 2021** | STEMI | 80 | **DCB (38)** | 19 | 7 | 12 | NA | NA | NA | NA | NA | NA | NA |
|  |  |  | **DES (42)** | 22 | 8 | 12 | NA | NA | NA | NA | NA | NA | NA |
| **Wang et.al 2022** | STEMI | 184 | **DCB (92)** | 64 | 24 | 29 | 30.62±10.01 | 83.56±8.55 % | 18.12±5.9% | 3.31±0.56 | NA | 0.53±0.28 | 2.71±0.53 |
|  |  |  | **DES (92)** | 64 | 19 | 30 | 33.51±13.13 | 85.56±7.01 % | 14.61±5.0% | 3.43±0.48 | NA | 0.50±0.26 | 2.92±0.44 |
| **Gobic et.al 2017** | STEMI | 78 | **DCB (41)** | NA | NA | NA | NA | NA | NA | 2.61±0.49 | NA | NA | 2.39±0.38 |
|  |  |  | **DES (37)** | NA | NA | NA | NA | NA | NA | 3.04±0.46 | NA | NA | 2.75±0.48 |
| **DEB-AMI** | STEMI | 99 | **DCB (50)** | 24 | 13 | 13 | 18.7±13.1 | 92.4±11.4 % | 14.1±9.6 % | 2.84±0.41 | 2.50±0.49 | 0.20±0.30 | 2.50±0.49 |
|  |  |  | **DES (49)** | 18 | 11 | 20 | 16.8± 8.7 | 88.4±13.6 % | 12.2±8.6 % | 2.78±0.53 | 2.53±0.41 | 0.34±0.41 | 2.53±0.41 |
| **DISSOLVE** | SVD | 247 | **DCB (129)** | 29 | 67 | 33 | 12.3±5.5 | 69.2±11.4 % | 18.6±9.0 % | 2.20±0.26 | NA | 0.68±0.28 | 1.78±0.26 |
|  |  |  | **DES (118)** | 28 | 57 | 34 | 11.5±5.1 | 70.6±10.7 % | 10.7±5.5 % | 2.20±0.26 | NA | 0.65±0.26 | 2.10±0.21 |
| **RESTORE SVD China** | SVD | 230 | **DCB (116)** | 10 | 59 | 9 | 15.4±6.2 | 71.9±9.3% | 20.0±8.3% | 2.42±0.15 | 0.60±0.23 | 1.64±0.29 | 1.64±0.29 |
|  |  |  | **DES (114)** | 10 | 47 | 10 | 14.4±5.6 | 68.5±9.2% | 15.42±6.9% | 2.42±0.18 | 0.62±0.22 | 0.62±0.22 | 1.99±0.28 |
| **PICCOLETO II** | SVD | 232 | **DCB (118)** | 47 | 44 | 27 | 13.5±7.3 | 75±17 % | 21.4±22% | 2.23±0.4 | NA | 0.82±0.5 | 1.89±0.3 |
|  |  |  | **DES (114)** | 44 | 35 | 34 | 14.0±6.9 | 76±15 % | 13.1±18% | 2.18±0.4 | NA | 0.83±0.4 | 2.29±0.4 |
| **PICCOLETO I** | SVD | 57 | **DCB (28)** | 15 | 5 | 8 | 12.41±5.89 | 86.0±12.1 % | 19±17.3% | 2.45±0.28 | NA | 0.48±0.33 | 2.47±0.22 |
|  |  |  | **DES (29)** | 15 | 3 | 11 | 11.38±7.12 | 89.14±10.6 % | 9.9± 9.2% | 2.36±0.25 | NA | 0.40±0.30 | 2.63±0.23 |
| **BASKET-SMALL 2** | SVD | 758 | **DCB (382)** | 128 | 179 | 75 | NA | NA | NA | NA | NA | NA | NA |
|  |  |  | **DES (276)** | 116 | 183 | 77 | NA | NA | NA | NA | NA | NA | NA |
| **BELLO** | SVD | 182 | **DCB (90)** | 10 | 10 | 8 | 15.32±7.45 | 72.14±10.05% | 29.84±10.24% | 2.15±0.27 | 1.56±0.32 | 0.60±0.24 | 1.47±0.30 |
|  |  |  | **DES (92)** | 12 | 16 | 9 | 14.4±5.6 | 83.3±8.7 % | 15.42± 6.92 % | 2.26±0.24 | 1.99±0.28 | 0.62±0.22 | 1.69±0.36 |

*DCB: Drug-coated balloon, DES: Drug-eluting stent, DS: Diameter stenosis, ISR: In-stent restenosis, MLD: Minimal lumen diameter, NA: Not assessed; PCB: Paclitaxel-coated balloon, PES: Paclitaxel-eluting stent, POBA: Plain old balloon angioplasty, SES: Sirolimus-eluting stent, STEMI: ST-segment elevation myocardial infarction, RVD: Reference vessel diameter.*

Supplemental Table 4. Types, brands, and drugs of devices used in each trial

| **Study** | **Group** | **Latest follow-up** | **Arms** | **DCB Drug** | **DCB Brand** | **DCB Generation** | **DES Drug** | **DES Brand** | **DES generation** |
| --- | --- | --- | --- | --- | --- | --- | --- | --- | --- |
| **ISAR-DESIRE 3** | ISR | 10 years | DCB vs DES vs POBA | Paclitaxel coated balloon | SeQuent Please | 2^nd^ generation | Paclitaxel eluting stent | Taxus Liberté | 2^nd^ generation |
| **RIBS IV** | ISR | 3 years | DCB vs DES | Paclitaxel coated balloon | SeQuent Please | 2^nd^ generation | Everolimus eluting stent | Xience Prime | 2^nd^ generation |
| **RIBS V** | ISR | 3 years | DCB vs DES | Paclitaxel coated balloon | SeQuent Please | 2^nd^ generation | Everolimus eluting stent | Xience Prime | 2^nd^ generation |
| **TIS** | ISR | 3 years | DCB vs DES | Paclitaxel coated balloon | SeQuent Please | 2^nd^ generation | Everolimus eluting stent | platinum-chromium Promus Element | 2^nd^ generation |
| **PEPCAD** | ISR | 3 years | DCB vs DES | Paclitaxel coated balloon | SeQuent Please | 2^nd^ generation | Paclitaxel eluting stent | Taxus Liberté | 2^nd^ generation |
| **PEPCAD CHINA** | ISR | 2 years | DCB vs DES | Paclitaxel coated balloon | SeQuent Please | 2^nd^ generation | Paclitaxel eluting stent | Taxus Liberté | 2^nd^ generation |
| **BIOLUX** | ISR | 18 months | DCB vs DES | Paclitaxel coated balloon | Pantera LUX | 2nd generation | Sirolimus eluting stent | Orsiro | 3^rd^ generation |
| **DARE** | ISR | 1 year | DCB vs DES | Paclitaxel coated balloon | SeQuent Please | 2nd generation | Everolimus eluting stent | Xience Prime | 2^nd^ generation |
| **RESTORE ISR** | ISR | 1 year | DCB vs DES | Paclitaxel coated balloon | SeQuent Please | 2nd generation | Everolimus eluting stent | Xience Prime | 2^nd^ generation |
| **SEDUCE** | ISR | 1 year | DCB vs DES | Paclitaxel coated balloon | SeQuent Please | 2nd generation | Everolimus eluting stent | Xience Prime | 2^nd^ generation |
| **REVELATION** | STEMI | 5 years | DCB vs DES | Paclitaxel coated balloon | Pantera Lux | 2^nd^ generation | Hybrid or everolimus eluting stent | Orsiro or Xience | 3^rd^ generation |
| **Hao et al.** | STEMI | 1 year | DCB vs DES | Paclitaxel coated balloon | Yinyi (Liaoning) Biotech Bingo | First generation | Paclitaxel eluting stent | Taxus Liberté | 2^nd^ generation |
| **Wang et al.** | STEMI | 1 year | DCB vs DES | Paclitaxel coated balloon | VasoguardTM | 2^nd^ generation | Sirolimus eluting stent | CordimaxTM | 2^nd^ generation |
| **Gobic et.al** | STEMI | 6 months | DCB vs DES | Paclitaxel coated balloon | SeQuent Please | 2^nd^ generation | Cobalt-chromium sirolimus eluting stent | Biomime | 3^rd^ generation |
| **DEB-AMI** | STEMI | 6 months | DCB vs DES | Paclitaxel coated balloon | DIOR | 2^nd^ generation | Paclitaxel eluting stent | Taxus Liberté | 2^nd^ generation |
| **DISSOLVE SVD** | SVD | 1 year | DCB vs DES | Paclitaxel coated balloon | Dissolve | 2^nd^ generation | zotarolimus eluting stent | Endeavor Resolute | 2^nd^ generation |
| **RESTORE SVD china** | SVD | 2 years | DCB vs DES | Paclitaxel coated balloon | Restore (cardinovum) | 3^rd^ generation | zotarolimus eluting stent | Resolute integrity | 2^nd^ generation |
| **PICCOLETO II** | SVD | 3 years | DCB vs DES | Paclitaxel coated balloon | Elutax SV | 2^nd^ generation | Everolimus eluting stent | Xience | 2^nd^ generation |
| **BASKET-SMALL II** | SVD | 3 years | DCB vs DES | Paclitaxel coated balloon | SeQuent Please | 2^nd^ generation | Everolimus eluting stent or Paclitaxel eluting stent | Xience or Taxus | 2^nd^ generation |
| **BELLO** | SVD | 2 years | DCB vs DES | Paclitaxel coated balloon | In.pact Falcon | 2^nd^ generation | Paclitaxel eluting stent | Taxus Liberte | 2^nd^ generation |
| **PICCOLETO** | SVD | 9 months | DCB vs DES | Paclitaxel coated balloon | Dior | 2^nd^ generation | Paclitaxel eluting stent | Taxus Liberte | 2^nd^ generation |

DCB: Drug-coated balloon, DES: Drug-eluting stent, ISR: In-stent restenosis, STEMI: ST-segment elevation myocardial infarction, SVD: Small vessel disease, POBA: Plain old balloon angioplasty

# Quality Assessment

Supplemental Figure 1. Risk of bias assessment of the included RCTs using the Cochrane risk-of-bias tool for randomized trials (RoB-2)


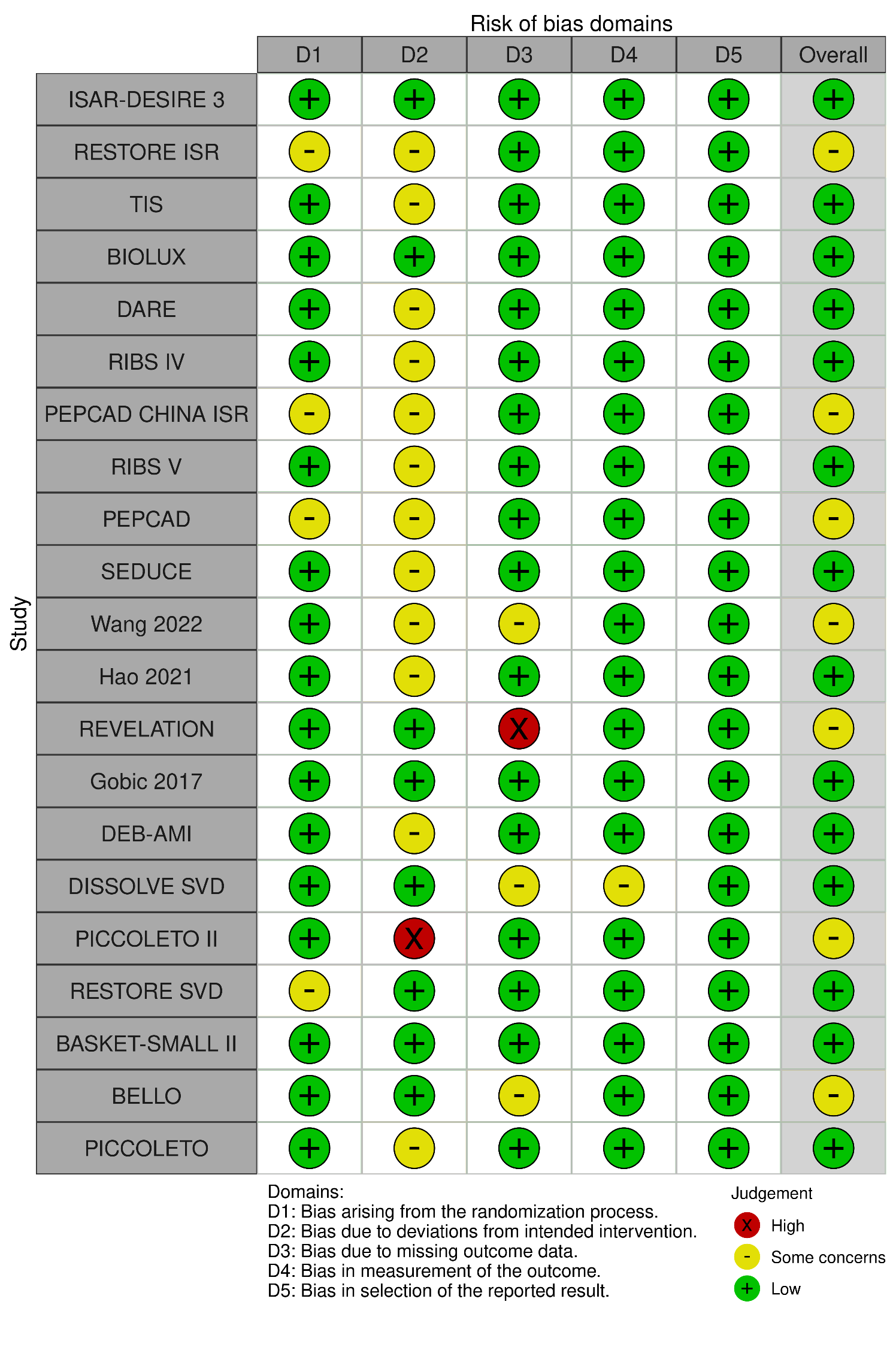


Supplemental Table 5. Quality of the reported outcomes using the Grading of Recommendations, Assessment, Development, and Evaluations (GRADE) framework

| **Outcome** | **NO. of Studies** | **Study Design** | **Quality of Evidence** | **Plausible**  **confounding** | **Overall Quality** | **Summary of Findings OR or MD (95%CI)** |
| --- | --- | --- | --- | --- | --- | --- |
| **Meta-Analysis of DCB vs DES in ISR** | | | | | | |
| **Target Lesion Revascularization (1-year)** | 10 | RCT | **Risk of Bias**:  Not Serious I**nconsistency**: Not Serious **Indirectness**:  Not Serious **Imprecision**:  Not Serious **Publication Bias**: Not Serious | Not Plausible | High | **DCB vs DES:**  1.36 (0.86-2.14) |
| **Target Lesion Revascularization (>1-year)** | 7 | RCT | **Risk of Bias**:  Not Serious I**nconsistency**: Serious **Indirectness**:  Not Serious **Imprecision**: Not Serious  **Publication Bias**: Not Serious | Not Plausible | Moderate | **DCB vs DES:**  1.23 (0.79-1.93) |
| **All-cause mortality (1-year)** | 9 | RCT | **Risk of Bias**:  Not Serious I**nconsistency**: Not Serious **Indirectness**:  Not Serious **Imprecision**:  Not Serious **Publication Bias**: Not Serious | Not Plausible | High | **DCB vs DES:**  0.71 (0.35-1.45) |
| **All-cause Mortality (>1-year)** | 6 | RCT | **Risk of Bias**:  Not Serious I**nconsistency**: Not Serious **Indirectness**:  Not Serious **Imprecision**:  Not Serious **Publication Bias**: Not Serious | Not Plausible | High | **DCB vs DES:** 0.90 (0.53-1.54) |
| **Cardiovascular Mortality (1-year)** | 10 | RCT | **Risk of Bias**:  Not Serious I**nconsistency**: Not Serious **Indirectness**:  Not Serious **Imprecision**:  Not Serious **Publication Bias**: Not Serious | Not Plausible | High | **DCB vs DES:** 0.90 (0.40-2.06) |
| **Cardiovascular Mortality (>1-year)** | 7 | RCT | **Risk of Bias**:  Not Serious I**nconsistency**: Not Serious **Indirectness**:  Not Serious **Imprecision**:  Not Serious **Publication Bias**: Not Serious | Not Plausible | High | **DCB vs DES:** 0.79 (0.51-1.22) |
| **Myocardial Infarction (1-year)** | 10 | RCT | **Risk of Bias**:  Not Serious I**nconsistency**: Not Serious **Indirectness**:  Not Serious **Imprecision**:  Not Serious **Publication Bias**:  Not Serious | Not Plausible | High | **DCB vs DES:**  0.79 (0.46-1.36) |
| **Myocardial Infarction (>1-year)** | 7 | RCT | **Risk of Bias**:  Not Serious I**nconsistency**: Not Serious **Indirectness**:  Not Serious **Imprecision**: Not Serious  **Publication Bias**:  Not Serious | Not Plausible | High | **DCB vs DES:**  1.02 (0.62-1.66) |
| **Thrombosis (1-year)** | 8 | RCT | **Risk of Bias**:  Not Serious I**nconsistency**: Not Serious **Indirectness**:  Not Serious **Imprecision**:  Not Serious **Publication Bias**:  Not Serious | Not Plausible | High | **DCB vs DES:**  0.88 (0.33-2.32) |
| **Thrombosis (>1-year)** | 4 | RCT | **Risk of Bias:**  Not Serious **Inconsistency:** Not Serious **Indirectness:**  Not Serious **Imprecision:**  Not Serious **Publication Bias:**  Not Serious | Not Plausible | High | **DCB vs DES:**  0.84 (0.27-2.57) |
| **Late lumen loss (6-months)** | 10 | RCT | **Risk of Bias**:  Not Serious I**nconsistency**: Not Serious **Indirectness**:  Not Serious **Imprecision**: Serious **Publication Bias**:  Not Serious | Not Plausible | Moderate | **DCB vs DES:**  -0.12 (-0.30; 0.06) |
| **Meta-Analysis of DCB vs DES in STEMI Patients** | | | | | | |
| **Target Lesion Revascularization** | 5 | RCT | **Risk of Bias**:  Not Serious I**nconsistency**: Not Serious **Indirectness**:  Not Serious **Imprecision**: Serious **Publication Bias**:  Not Serious | Not Plausible | Moderate | **DCB vs DES:**  2.08 (0.75-5.76) |
| **All-cause Mortality** | 4 | RCT | **Risk of Bias**:  Not Serious I**nconsistency**: Not Serious **Indirectness**:  Not Serious **Imprecision**: Serious **Publication Bias**:  Not Serious | Not Plausible | Moderate | **DCB vs DES:**  1.04 (0.25-4.25) |
| **Cardiovascular Mortality** | 5 | RCT | **Risk of Bias**:  Not Serious I**nconsistency**: Not Serious **Indirectness**:  Not Serious **Imprecision**: Serious **Publication Bias**:  Not Serious | Not Plausible | Moderate | **DCB vs DES:**  0.85 (0.22-3.31) |
| **Myocardial Infarction** | 5 | RCT | **Risk of Bias**:  Not Serious I**nconsistency**: Not Serious **Indirectness**:  Not Serious **Imprecision**: Serious **Publication Bias**:  Not Serious | Not Plausible | Moderate | **DCB vs DES:**  1.18 (0.44-3.14) |
| **Thrombosis** | 3 | RCT | **Risk of Bias**:  Not Serious I**nconsistency**: Not Serious **Indirectness**:  Not Serious **Imprecision**: Serious **Publication Bias**:  Not Serious | Not Plausible | Moderate | **DCB vs DES:**  1.12 (0.25-4.96) |
| **Late Lumen Loss (6 months)** | 4 | RCT | **Risk of Bias**:  Not Serious I**nconsistency**: Serious **Indirectness**:  Not Serious **Imprecision**: Serious **Publication Bias**:  Not Serious | Not Plausible | Low | **DCB vs DES:**  **-**0.20 (-1.16; 0.76) |
| **Meta-Analysis of DCB vs DES in Small Vessel Disease (SVD) Patients** | | | | | | |
| **Target Lesion Revascularization (1-year)** | 6 | RCT | **Risk of Bias**:  Not Serious I**nconsistency**: Not Serious **Indirectness**:  Not Serious **Imprecision**: Serious **Publication Bias**:  Not Serious | Not Plausible | Moderate | **DCB vs DES:**  1.37 (0.81-2.30) |
| **Target Lesion Revascularization (>1-year)** | 4 | RCT | **Risk of Bias**:  Not Serious I**nconsistency**: Not Serious **Indirectness**:  Not Serious **Imprecision**: Serious **Publication Bias**:  Not Serious | Not Plausible | Moderate | **DCB vs DES:**  0.85 (0.52-1.38) |
| **All-cause mortality (1-year)** | 6 | RCT | **Risk of Bias**:  Not Serious I**nconsistency**: Not Serious **Indirectness**:  Not Serious **Imprecision**: Serious **Publication Bias**:  Not Serious | Not Plausible | Moderate | **DCB vs DES:** 0.58 (0.29-1.16) |
| **All-cause Mortality (>1-year)** | 4 | RCT | **Risk of Bias**:  Not Serious I**nconsistency**: Not Serious **Indirectness**:  Not Serious **Imprecision**: Serious **Publication Bias**:  Not Serious | Not Plausible | Moderate | **DCB vs DES:**  0.92 (0.56-1.51) |
| **Cardiovascular Mortality (1-year)** | 6 | RCT | **Risk of Bias**:  Not Serious I**nconsistency**: Not Serious **Indirectness**:  Not Serious **Imprecision**: Serious **Publication Bias**:  Not Serious | Not Plausible | Moderate | **DCB vs DES:**  0.47 (0.19-1.16) |
| **Cardiovascular Mortality (>1-year)** | 4 | RCT | **Risk of Bias**:  Not Serious I**nconsistency**: Not Serious **Indirectness**:  Not Serious **Imprecision**: Serious **Publication Bias**:  Not Serious | Not Plausible | Moderate | **DCB vs DES:**  0.78 (0.40-1.54) |
| **Myocardial Infarction (1-year)** | 6 | RCT | **Risk of Bias**:  Not Serious I**nconsistency**: Not Serious **Indirectness**:  Not Serious **Imprecision**: Serious **Publication Bias**:  Not Serious | Not Plausible | Moderate | **DCB vs DES:**  1.04 (0.39-2.74) |
| **Myocardial Infarction (>1-year)** | 4 | RCT | **Risk of Bias**:  Not Serious I**nconsistency**: Not Serious **Indirectness**:  Not Serious **Imprecision**: Serious **Publication Bias**:  Not Serious | Not Plausible | Moderate | **DCB vs DES:**  0.66 (0.29-1.52) |
| **Thrombosis (1-year)** | 5 | RCT | **Risk of Bias**:  Not Serious I**nconsistency**: Not Serious **Indirectness**:  Not Serious **Imprecision**: Serious **Publication Bias**:  Not Serious | Not Plausible | Moderate | **DCB vs DES:**  1.08 (0.31-3.76) |
| **Thrombosis (>1-year)** | 3 | RCT | **Risk of Bias**:  Not Serious I**nconsistency**: Serious **Indirectness**:  Not Serious **Imprecision**: Serious **Publication Bias**:  Not Serious | Not Plausible | Low | **DCB vs DES:**  0.86 (0.10-7.50) |
| **Late lumen loss (6-months)** | 3 | RCT | **Risk of Bias**:  Not Serious I**nconsistency**: Not Serious **Indirectness**:  Not Serious **Imprecision**:  Not Serious **Publication Bias**:  Not Serious | Not Plausible | High | **DCB vs DES:**  -0.38 (-0.53; -0.22) |

# Main Results of In-Stent Restenosis (ISR)

**Supplemental Figure 2.** Forest plots representing the risk of A. 1-year all-cause mortality in ISR patients B. >1-year all-cause mortality in ISR patients


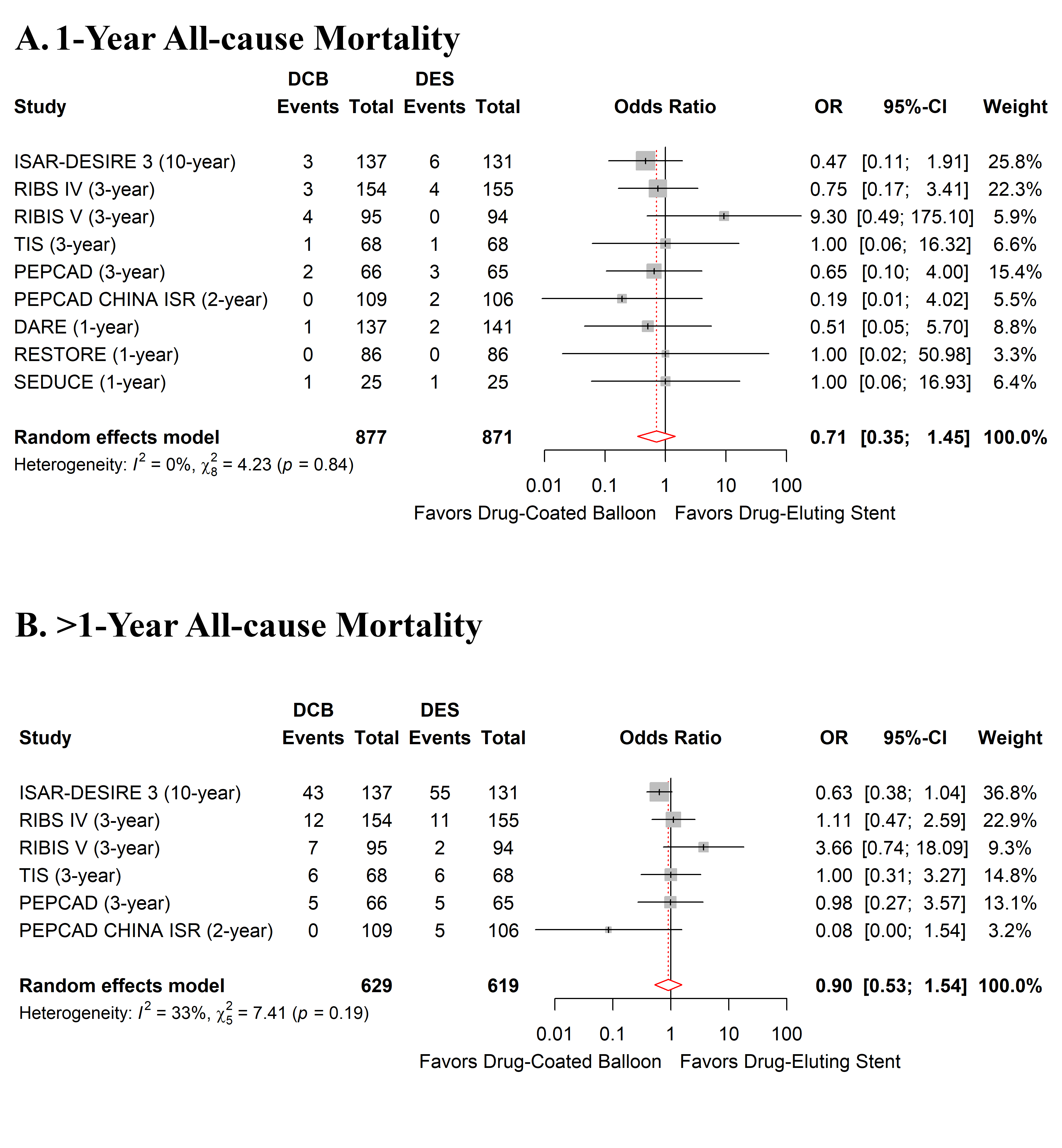


**Supplemental Figure 3.** Forest plots representing the risk of A. 1-year cardiovascular mortality in ISR patients B. >1-year cardiovascular mortality in ISR patients


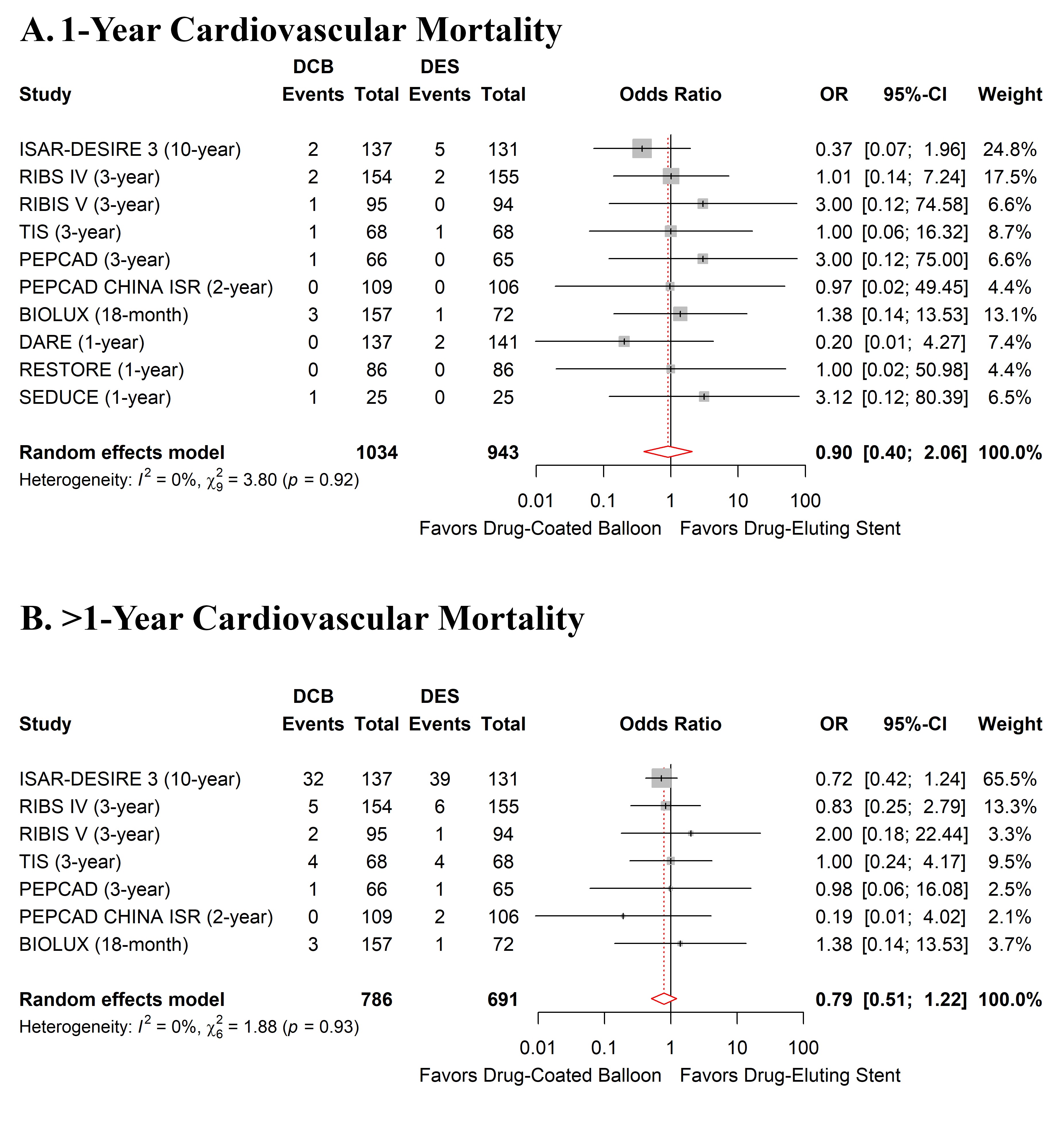


**Supplemental Figure 4**. Forest plots representing the risk of A. 1-year myocardial infarction in ISR patients B. >1-year myocardial infarction in ISR patients


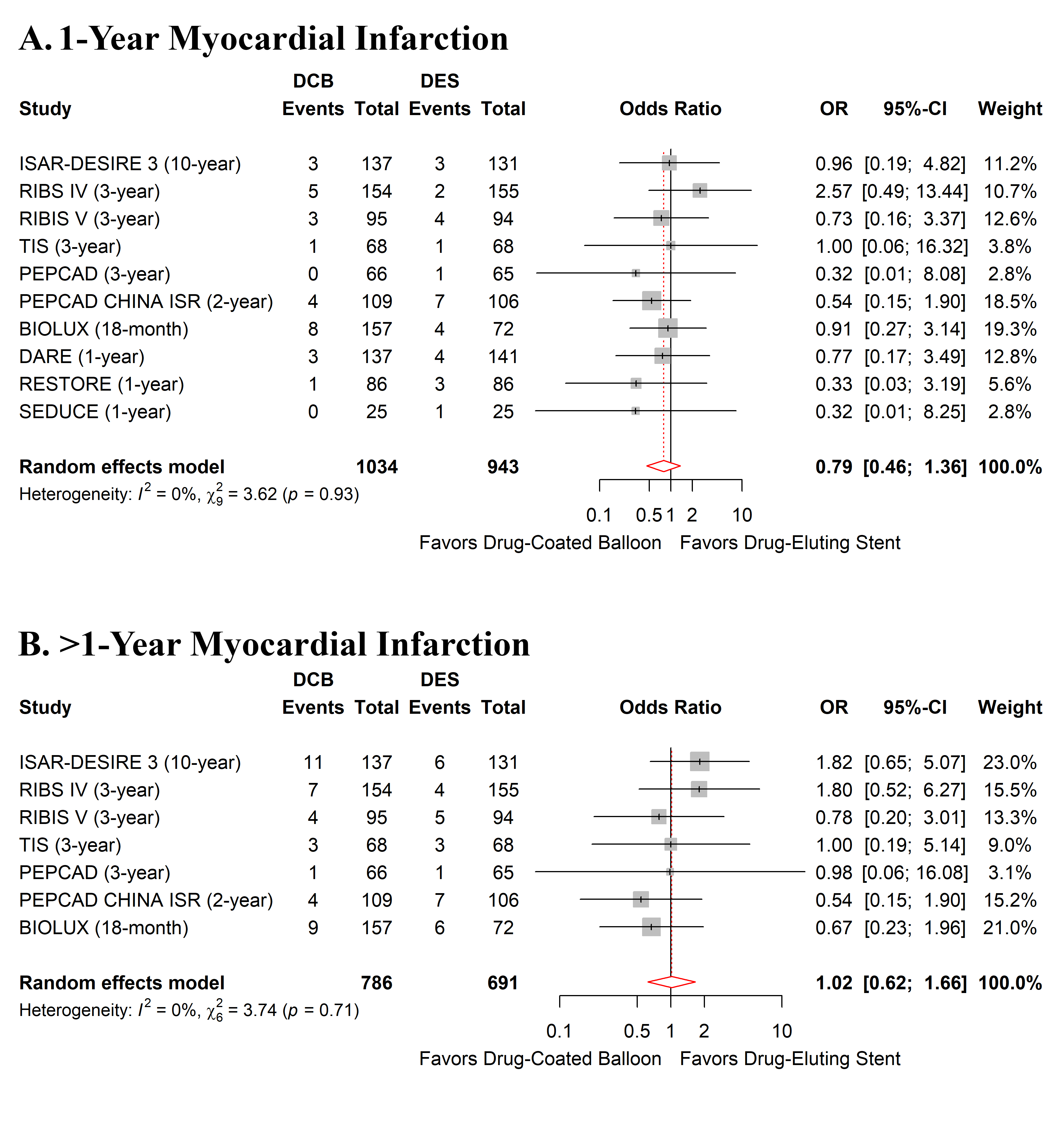


**Supplemental Figure 5**. Forest plots representing the risk of A. 1-year thrombosis in ISR patients B. >1-year thrombosis in ISR patients

**
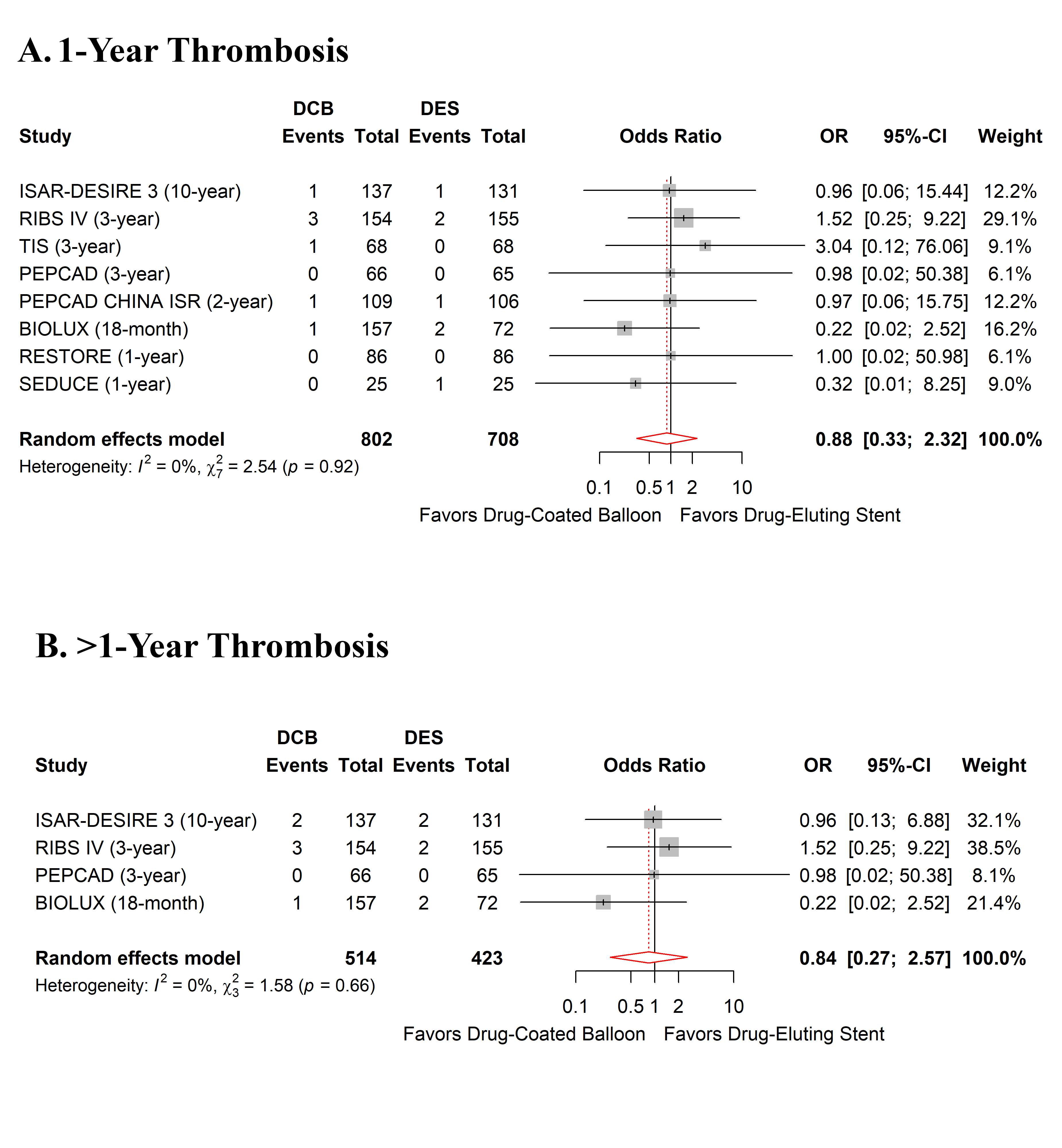
**

**Supplemental Figure 6.** Forest plots representing late lumen loss in ISR patients


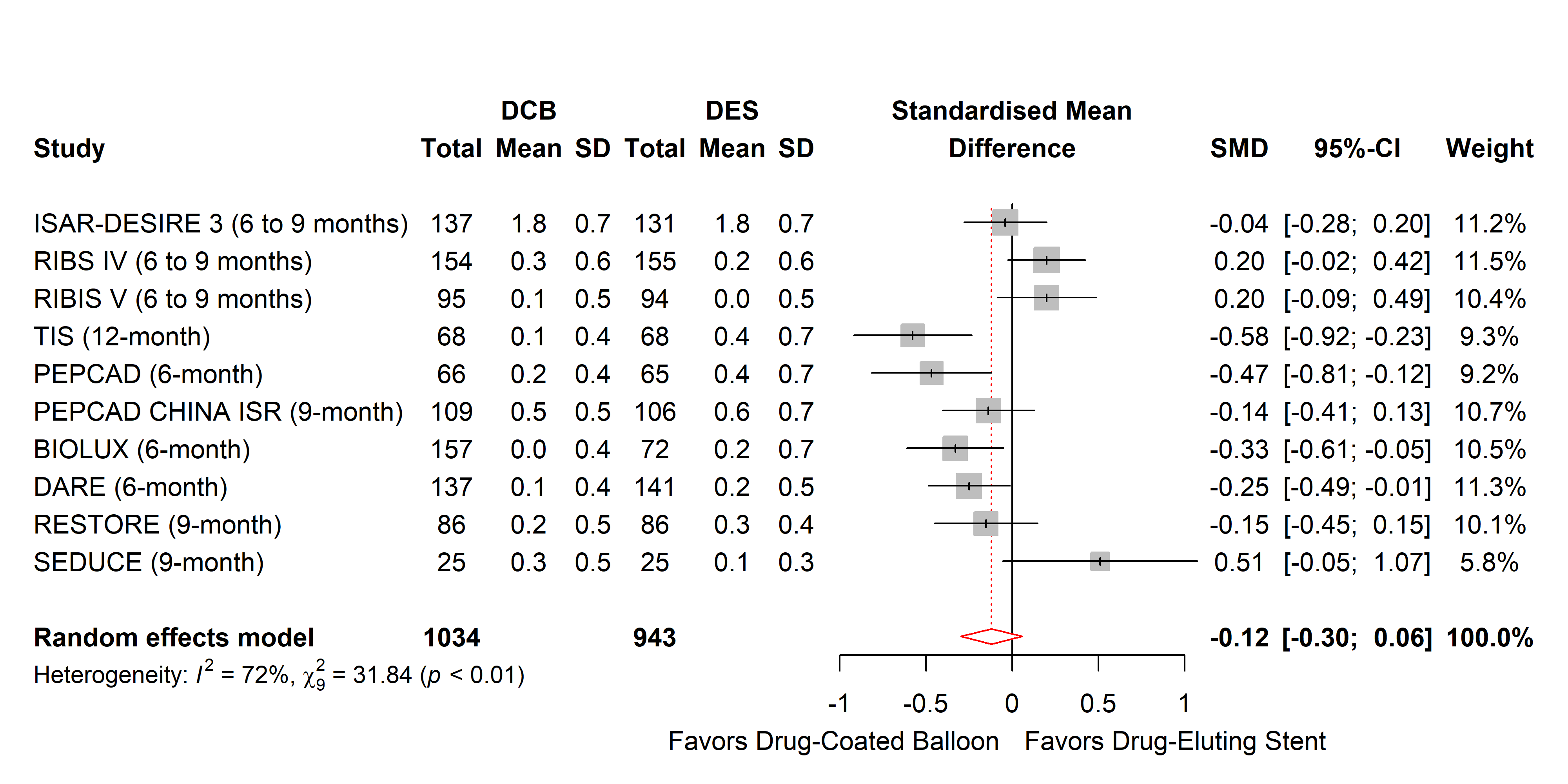


# Main Results of ST-Elevation Myocardial Infarction (STEMI)

**Supplemental Figure 7.** Forest plot demonstrating the risk of 1-year all-cause mortality in STEMI patients between DCB and DES

**
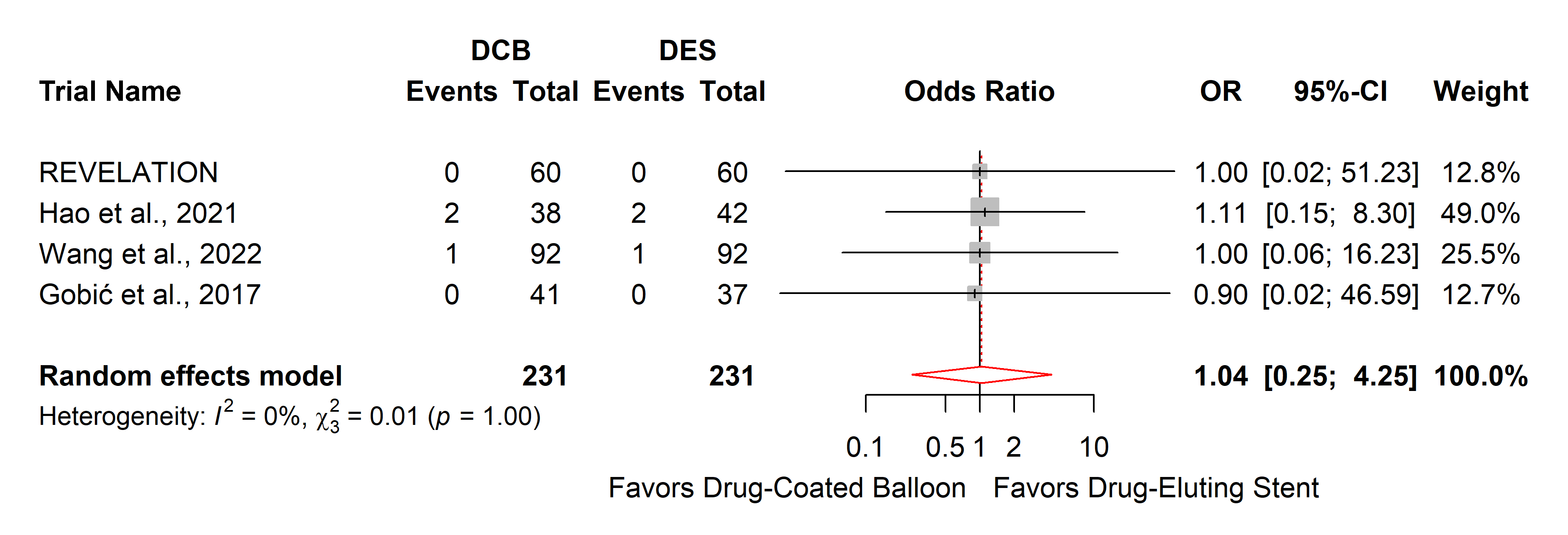
**

**Supplemental Figure 8**. Forest plot demonstrating the risk of 1-year cardiovascular mortality in STEMI patients between DCB and DES


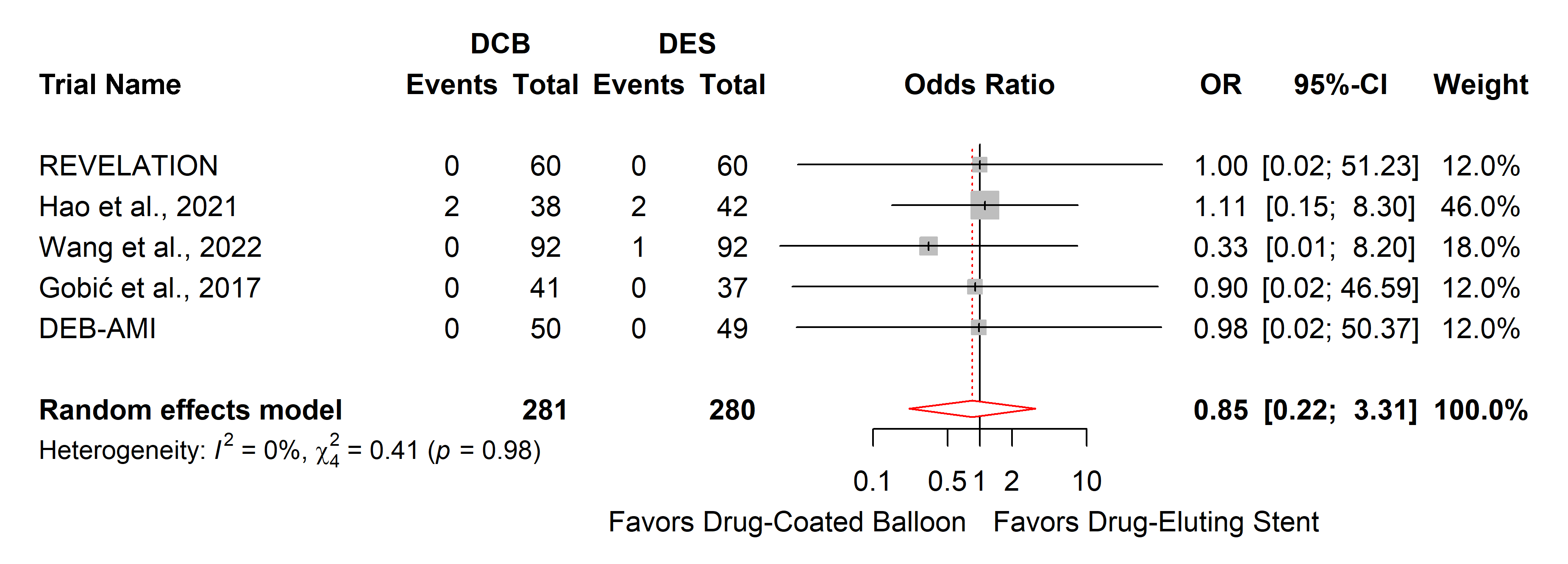


**Supplemental Figure 9.** Forest plot demonstrating the risk of 1-year myocardial infarction between DCB and DES


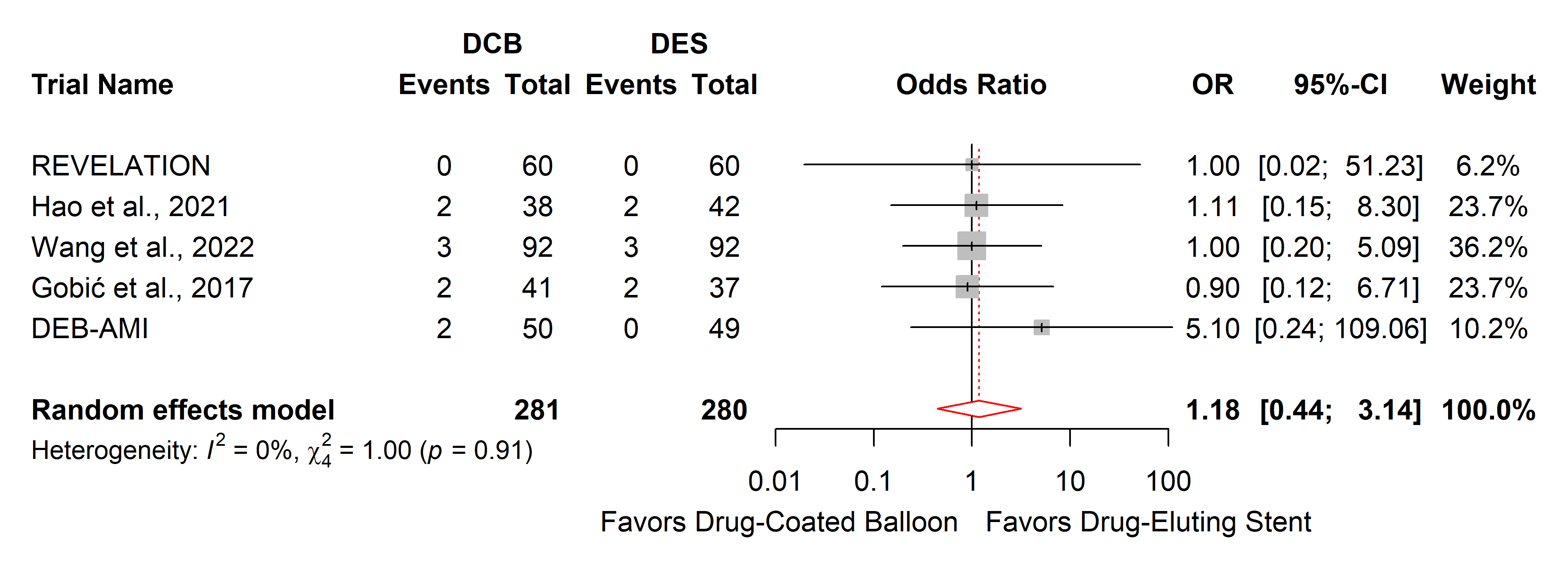


**Supplemental Figure 10**. Forest plot demonstrating the risk of 1-year Thrombosis between DCB and DES


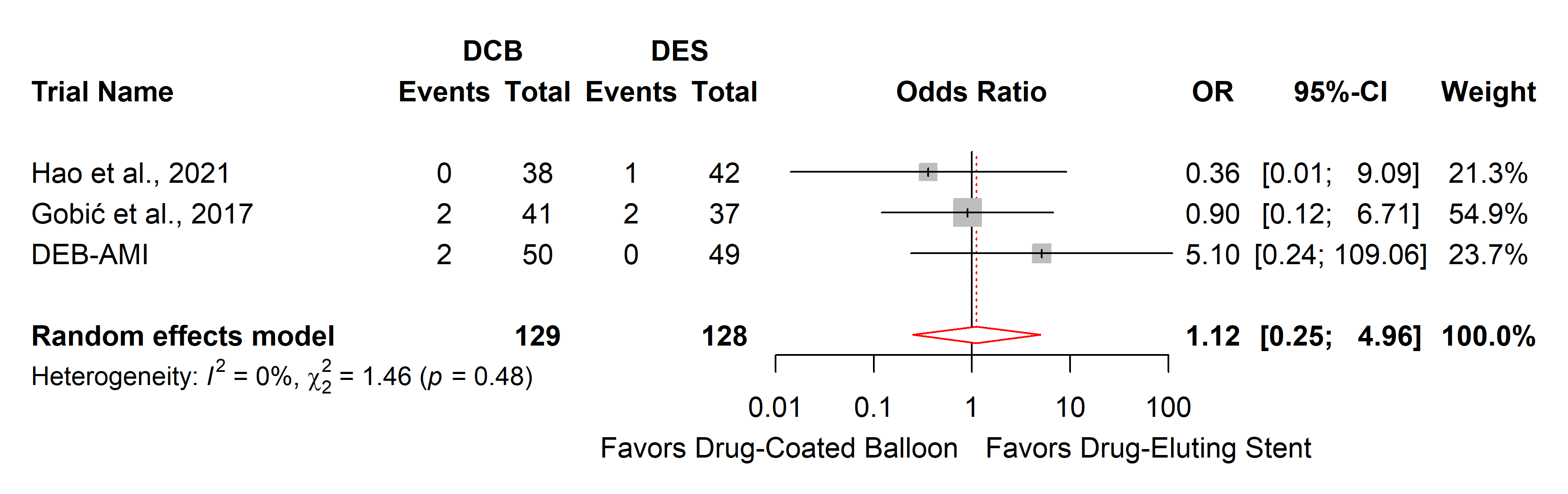


**Supplemental Figure 11**. Forest plot demonstrating the difference in 6-9 months late lumen loss between DCB and DES


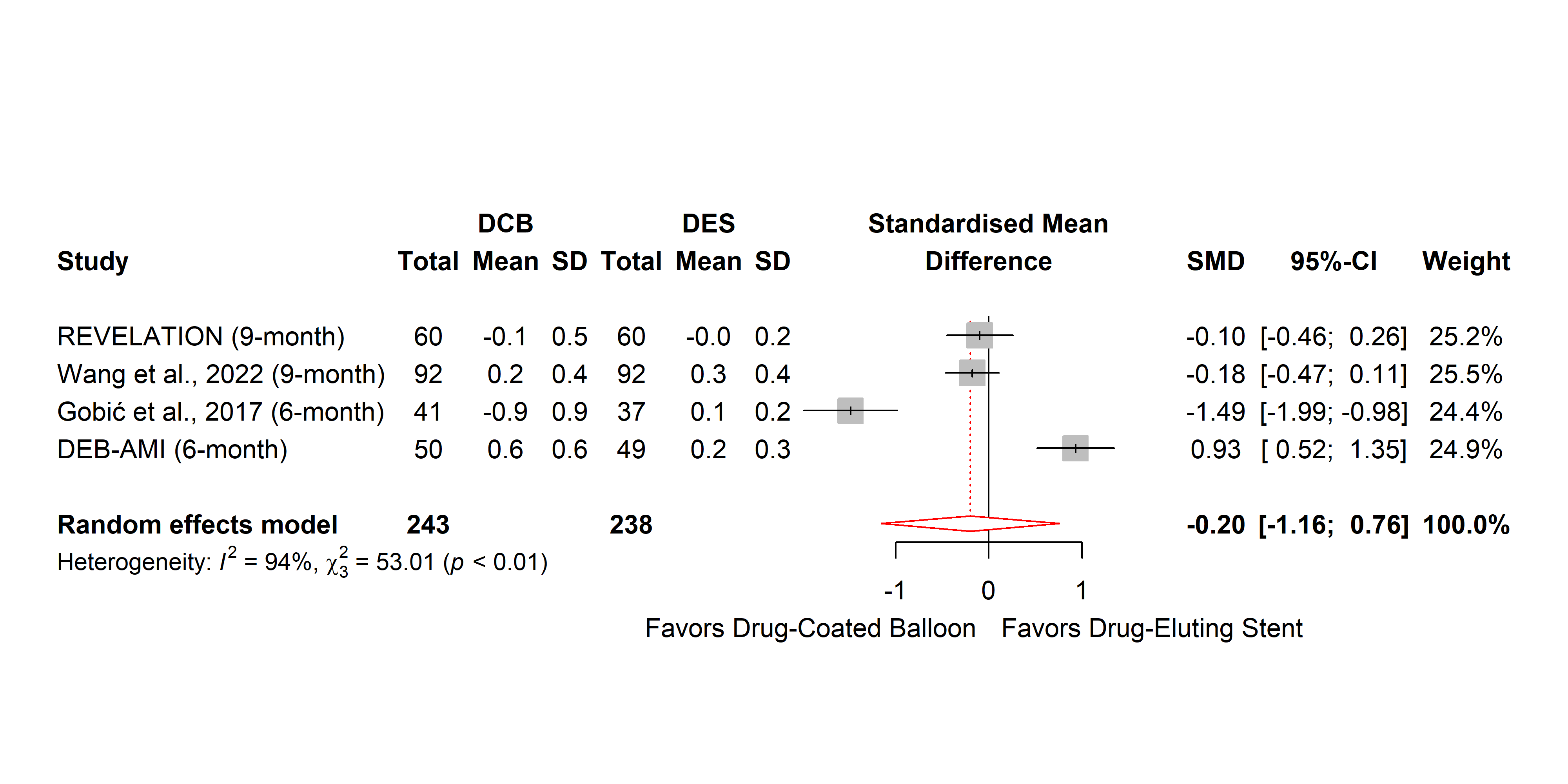


# Main Results of Small Vessel Disease (SVD)

**Supplemental Figure 12.** Forest plot representing the difference in 6-month late lumen loss between DCB and DES in SVD patients


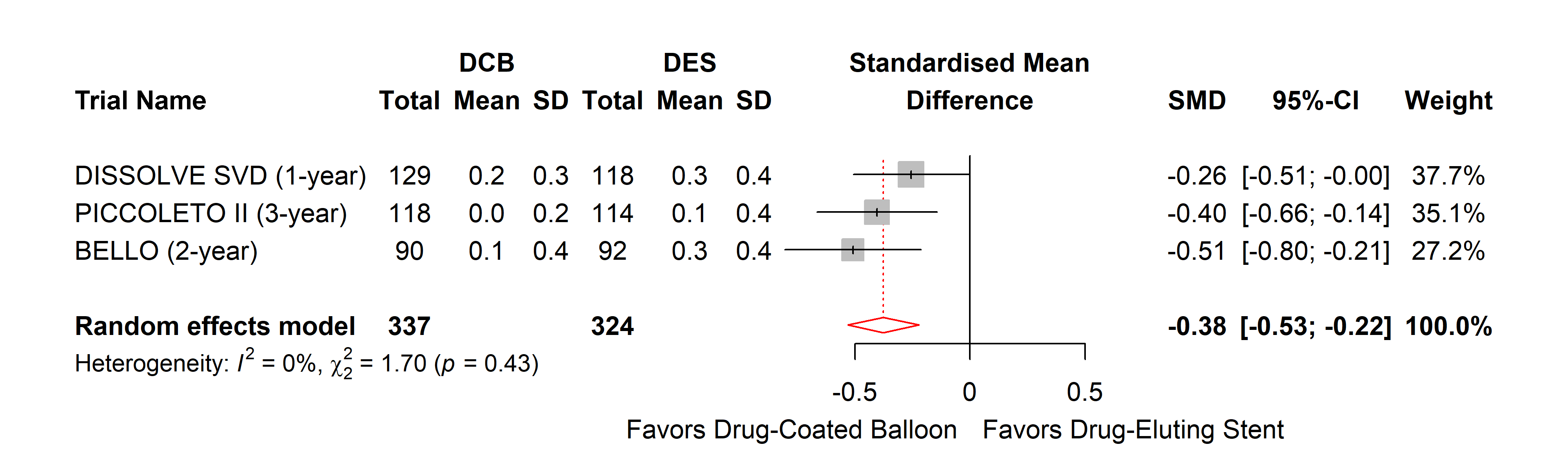


**Supplemental Figure 13**. Forest plots representing the risk of A. 1-year all-cause mortality in SVD patients B. >1-year all-cause mortality in SVD patients


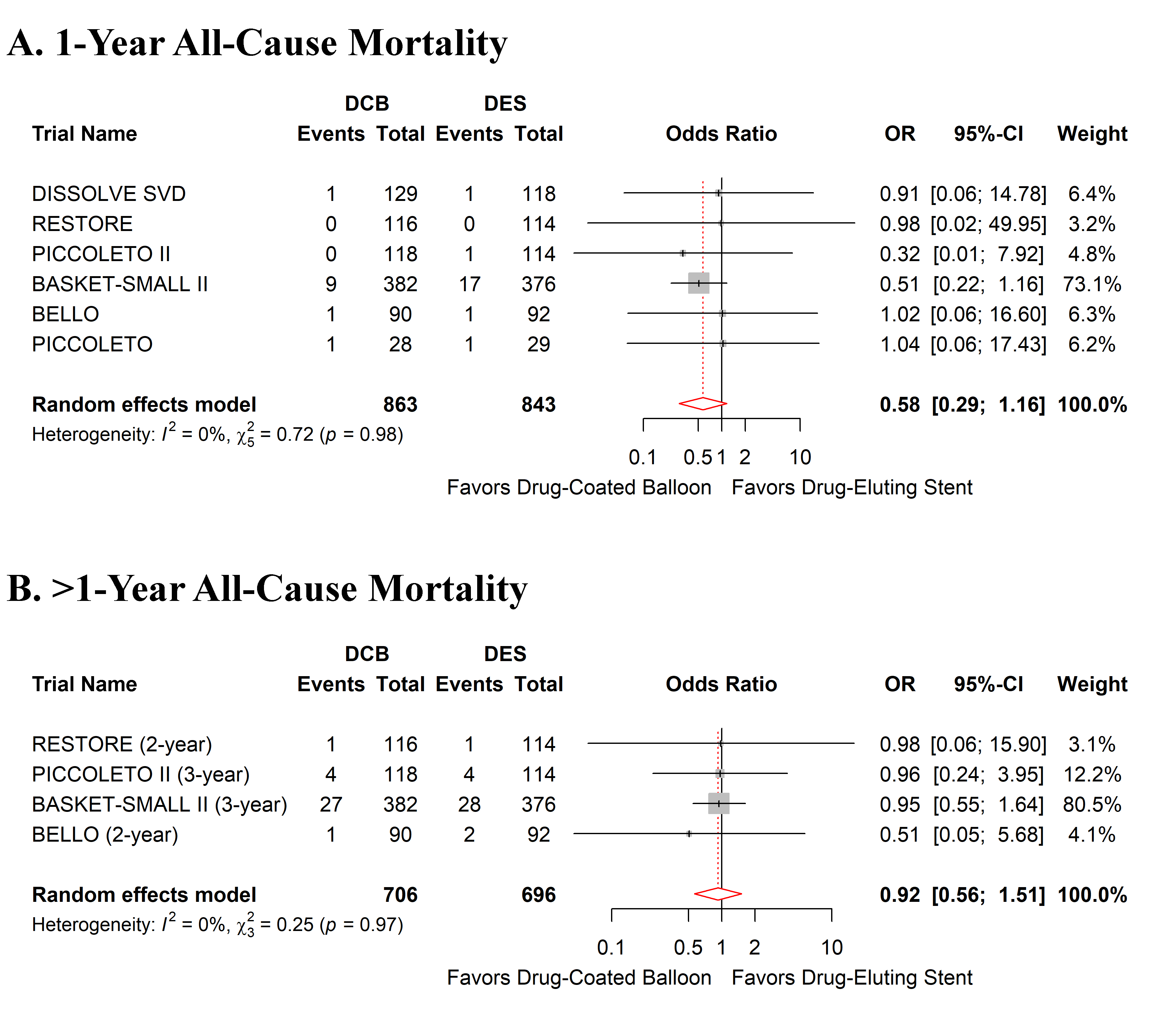


**Supplemental Figure 14.** Forest plot representing the risk of A. 1-year cardiovascular mortality in SVD patients B. >1-year cardiovascular mortality in SVD patients


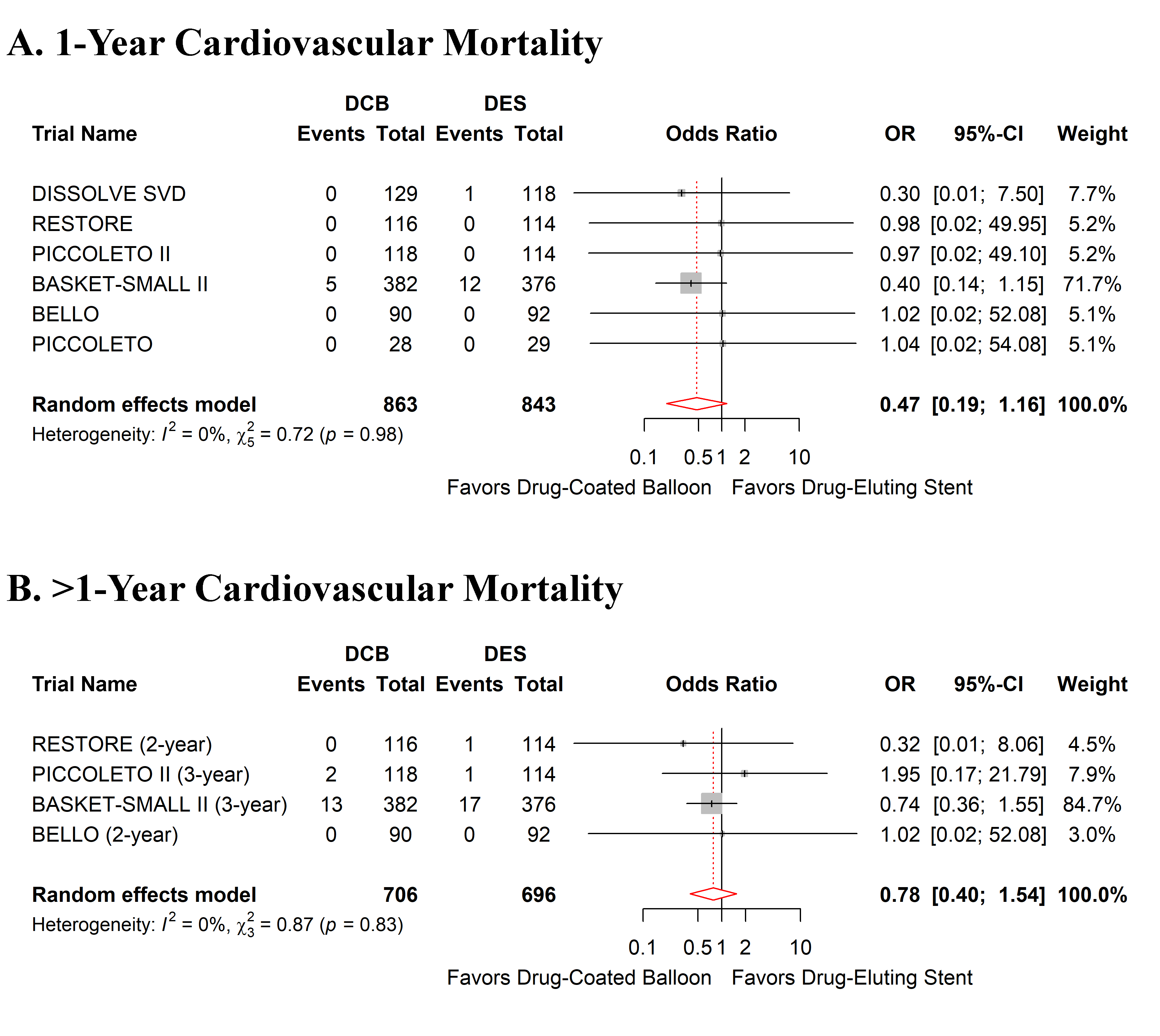


**Supplemental Figure 15**. Forest plot representing the risk of A. 1-year myocardial infarction in SVD patients B. >1-year myocardial infarction in SVD patients


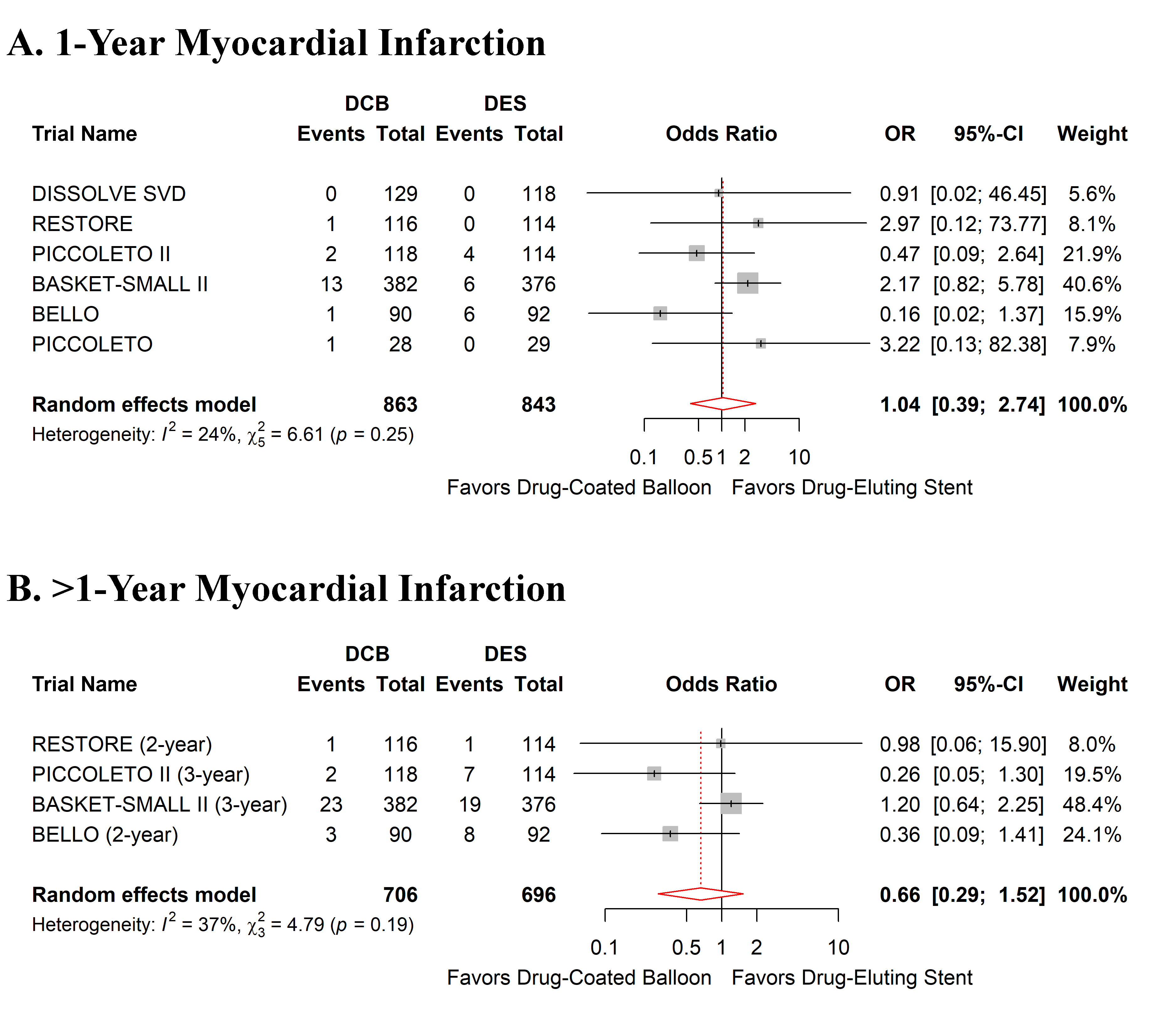


**Supplemental Figure 16.** Forest plot representing the risk of A. 1-year thrombosis in SVD patients B. >1-year thrombosis in SVD patients


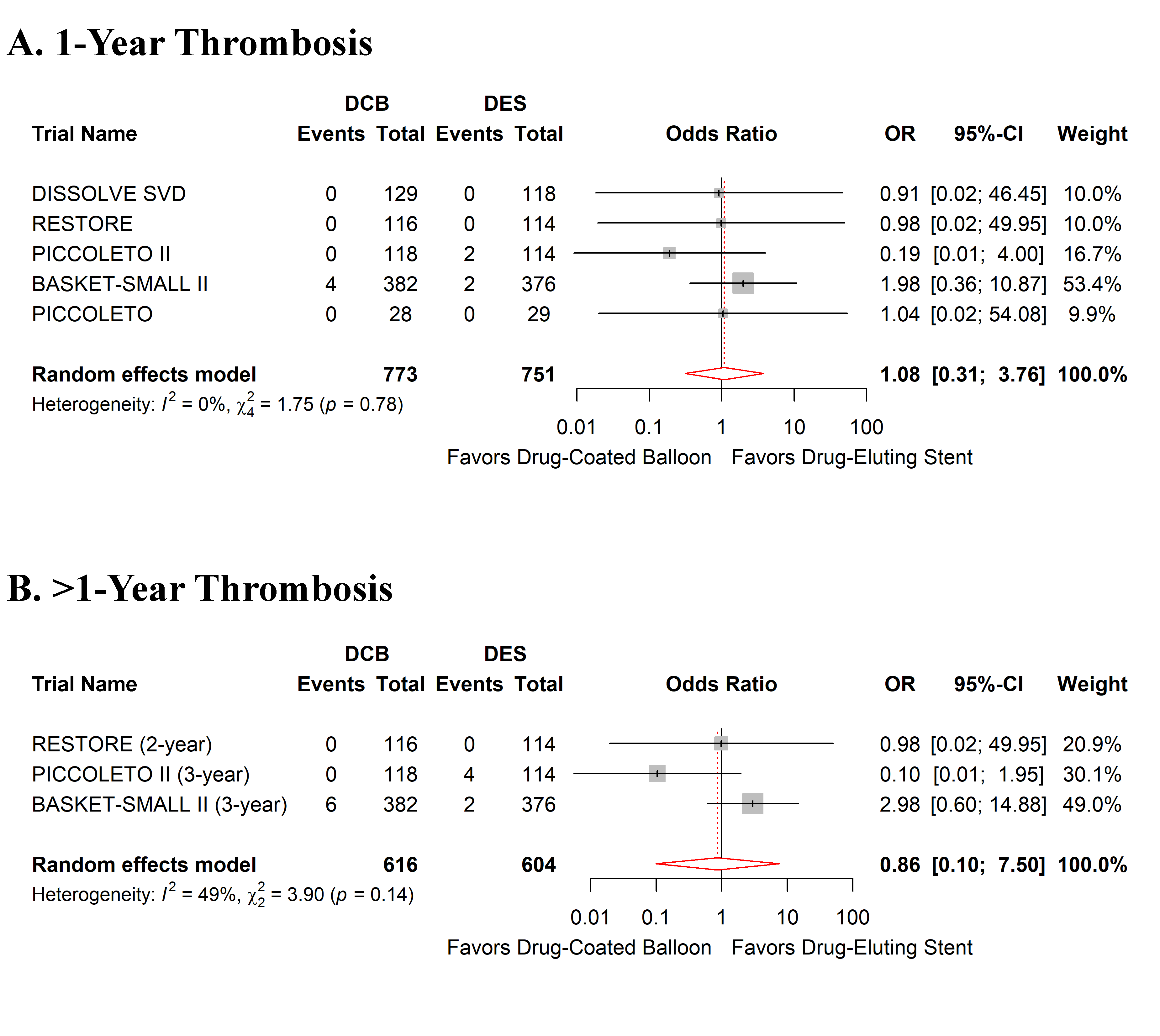


# Sensitivity Analysis

## Sensitivity Analysis in ISR Patients

**Supplemental Table 6.** Sensitivity analysis using leave-one-out method to assess each study's contribution to the pooled estimates in ISR

| **1-year Target lesion revascularization** | | | |
| --- | --- | --- | --- |
| **Excluded Study** | **Pooled OR (95%CI)** | **P-value** | **I²** |
| ISAR-DESIRE 3 | 1.26 (0.74-2.17) | 0.38 | 46.2% |
| RIBS IV | 1.19 (0.75-1.89) | 0.43 | 34.7% |
| RIBS V | 1.27 (0.81-2.01) | 0.29 | 42.6% |
| TIS | 1.55 (1.02-2.34) | 0.03 | 25.8% |
| PEPCAD | 1.50 (0.96-2.34) | 0.07 | 36.5% |
| PEPCAD CHINA ISR | 1.35 (0.79-2.32) | 0.26 | 49.5% |
| BIOLUX | 1.37 (0.81-2.32) | 0.23 | 49.2% |
| DARE | 1.36 (0.80-2.32) | 0.24 | 49.3% |
| RESTORE | 1.28 (0.80-2.04) | 0.28 | 44.5% |
| SEDUCE | 1.40 (0.87-2.24) | 0.16 | 47.1% |
| **>1-year Target lesion revascularization** | | | |
| **Excluded Study** | **Pooled OR (95%CI)** | **P-value** | **I²** |
| ISAR-DESIRE 3 (10-year) | 1.17 (0.65-2.12) | 0.58 | 55.2% |
| RIBS IV (3-year) | 1.08 (0.68-1.71) | 0.74 | 38.9% |
| RIBS V (3-year) | 1.14 (0.73-1.77) | 0.55 | 45.3% |
| TIS (3-year) | 1.41 (0.93-2.14) | 0.10 | 32% |
| PEPCAD (3-year) | 1.36 (0.87-2.11) | 0.16 | 42.2% |
| PEPCAD CHINA ISR (2-year) | 1.22 (0.70-2.12) | 0.46 | 56.1% |
| BIOLUX (18-month) | 1.25 ()0.73-2.12 | 0.40 | 55.6% |
| **1-year All-cause mortality** | | | |
| **Excluded Study** | **Pooled OR (95%CI)** | **P-value** | **I²** |
| ISAR-DESIRE 3 | 0.81 (0.35-1.87) | 0.63 | 0.0% |
| RIBS IV | 0.69 (0.30-1.56) | 0.38 | 0.0% |
| RIBS V | 0.60 (0.28-1.25) | 0.17 | 0.0% |
| TIS | 0.69 (0.32-1.44) | 0.32 | 0.0% |
| PEPCAD | 0.71 (0.33-1.56) | 0.40 | 0.0% |
| PEPCAD CHINA ISR | 0.76 (0.36-1.59) | 0.47 | 0.0% |
| BIOLUX | 0.70 (0.34-1.44) | 0.34 | 0.0% |
| DARE | 0.72 (0.34-1.54) | 0.40 | 0.0% |
| RESTORE | 0.69 (0.33-1.44) | 0.32 | 0.0% |
| SEDUCE | 0.69 (0.32-1.44) | 0.33 | 0.0% |
| **>1-year All-cause mortality** | | | |
| **Excluded Study** | **Pooled OR (95%CI)** | **P-value** | **I²** |
| ISAR-DESIRE 3 (10-year) | 1.10 (0.56-2.16) | 0.77 | 23.4% |
| RIBS IV (3-year) | 0.87 (0.43-1.76) | 0.70 | 41.1% |
| RIBS V (3-year) | 0.74 (0.50-1.09) | 0.13 | 0.0% |
| TIS (3-year) | 0.90 (0.46-1.75) | 0.76 | 45.1% |
| PEPCAD (3-year) | 0.90 (0.47-1.74) | 0.76 | 45.4% |
| PEPCAD CHINA ISR (2-year) | 0.92 (0.57-1.46) | 0.72 | 20.6% |
| **1-year Cardiovascular mortality** | | | |
| **Excluded Study** | **Pooled OR (95%CI)** | **P-value** | **I²** |
| ISAR-DESIRE 3 | 1.21 (0.46-3.13) | 0.69 | 0.0% |
| RIBS IV | 0.88 (0.35-2.19) | 0.78 | 0.0% |
| RIBS V | 0.83 (0.35-1.95) | 0.67 | 0.0% |
| TIS | 0.89 (0.37-2.12) | 0.80 | 0.0% |
| PEPCAD | 0.83 (0.35-1.95) | 0.67 | 0.0% |
| PEPCAD CHINA ISR | 0.90 (0.38-2.09) | 0.80 | 0.0% |
| BIOLUX | 0.84 (0.34-2.05) | 0.71 | 0.0% |
| DARE | 1.01 (0.43-2.40) | 0.96 | 0.0% |
| RESTORE | 0.89 (0.38-2.09) | 0.80 | 0.0% |
| SEDUCE | 0.82 (0.35-1.94) | 0.66 | 0.0% |
| **>1-year Cardiovascular mortality** | | | |
| **Excluded Study** | **Pooled OR (95%CI)** | **P-value** | **I²** |
| ISAR-DESIRE 3 (10-year) | 0.93 (0.44-1.97) | 0.85 | 0.0% |
| RIBS IV (3-year) | 0.77 (0.48-1.25) | 0.30 | 0.0% |
| RIBS V (3-year) | 0.76 (0.48-1.19) | 0.23 | 0.0% |
| TIS (3-year) | 0.76 (0.48-1.21) | 0.26 | 0.0% |
| PEPCAD (3-year) | 0.78 (0.50-1.22) | 0.28 | 0.0% |
| PEPCAD CHINA ISR (2-year) | 0.81 (0.51-1.26) | 0.35 | 0.0% |
| BIOLUX (18-month) | 0.76 (0.49-1.20) | 0.25 | 0.0% |
| **1-year Myocardial infarction** | | | |
| **Excluded Study** | **Pooled OR (95%CI)** | **P-value** | **I²** |
| ISAR-DESIRE 3 | 0.77 (0.43-1.36) | 0.37 | 0.0% |
| RIBS IV | 0.68 (0.38-1.21) | 0.19 | 0.0% |
| RIBS V | 0.79 (0.44-1.42) | 0.44 | 0.0% |
| TIS | 0.78 (0.45-1.35) | 0.38 | 0.0% |
| PEPCAD | 0.81 (0.46-1.40) | 0.45 | 0.0% |
| PEPCAD CHINA ISR | 0.86 (0.47-1.56) | 0.62 | 0.0% |
| BIOLUX | 0.76 (0.41-1.39) | 0.37 | 0.0% |
| DARE | 0.79 (0.44-1.41) | 0.43 | 0.0% |
| RESTORE | 0.83 (0.47-1.45) | 0.51 | 0.0% |
| SEDUCE | 0.81 (0.46-1.40) | 0.45 | 0.0% |
| **>1-year Myocardial infarction** | | | |
| **Excluded Study** | **Pooled OR (95%CI)** | **P-value** | **I²** |
| ISAR-DESIRE 3 (10-year) | 0.85 (0.48-1.49) | 0.58 | 0.0% |
| RIBS IV (3-year) | 0.91 (0.53-1.56) | 0.74 | 0.0% |
| RIBS V (3-year) | 1.05 (0.62-1.79) | 0.83 | 0.0% |
| TIS (3-year) | 1.01 (0.60-1.70) | 0.94 | 0.0% |
| PEPCAD (3-year) | 1.01 (0.61-1.67) | 0.94 | 0.0% |
| PEPCAD CHINA ISR (2-year) | 1.13 (0.66-1.94) | 0.63 | 0.0% |
| BIOLUX (18-month) | 1.13 (0.65-1.97) | 0.65 | 0.0% |
| **1-year Thrombosis** | | | |
| **Excluded Study** | **Pooled OR (95%CI)** | **P-value** | **I²** |
| ISAR-DESIRE 3 | 0.86 (0.30-2.44) | 0.78 | 0.0% |
| RIBS IV | 0.70 (0.22-2.22) | 0.54 | 0.0% |
| RIBS V | 0.87 (0.33-2.32) | 0.79 | 0.0% |
| TIS | 0.77 (0.31-2.37) | 0.78 | 0.0% |
| PEPCAD | 0.87 (0.32-2.88) | 0.95 | 0.0% |
| PEPCAD CHINA ISR | 0.86 (0.30-2.44) | 0.95 | 0.0% |
| BIOLUX | 1.14 (0.39-3.30) | 0.80 | 0.0% |
| DARE | 0.87 (0.33-2.32) | 0.79 | 0.0% |
| RESTORE | 0.87 (0.31-2.37) | 0.78 | 0.0% |
| SEDUCE | 0.96 (0.34-2.68) | 0.95 | 0.0% |
| **>1-year Thrombosis** | | | |
| **Excluded Study** | **Pooled OR (95%CI)** | **P-value** | **I²** |
| ISAR-DESIRE 3 (10-year) | 0.79 (0.20-3.06) | 0.73 | 0.0% |
| RIBS IV (3-year) | 0.57 (0.13-2.41) | 0.45 | 0.0% |
| PEPCAD (3-year) | 0.82 (0.25-2.65) | 0.75 | 0.0% |
| BIOLUX (18-month) | 1.20 (0.34-4.24) | 0.77 | 0.0% |
| **6-month Late lumen loss** | | | |
| **Excluded Study** | **Pooled SMD (95%CI)** | **P-value** | **I²** |
| ISAR-DESIRE 3 | -0.12 (-0.33; 0.07) | 0.21 | 74.6% |
| RIBS IV | -0.16 (-0.33; 0.01) | 0.06 | 65.4% |
| RIBS V | -0.15 (-0.33; 0.02) | 0.08 | 70.2% |
| TIS | -0.07 (-0.24; 0.09) | 0.37 | 66.9% |
| PEPCAD | -0.08 (-0.26; 0.09) | 0.35 | 70.9% |
| PEPCAD CHINA ISR | -0.11 (-0.32; 0.08) | 0.25 | 74.8% |
| BIOLUX | -0.09 (-0.28; 0.09) | 0.33 | 72.6% |
| DARE | -0.10 (-0.30; 0.09) | 0.31 | 73.6% |
| RESTORE | -0.11 (-0.31; 0.08) | 0.31 | 73.6% |
| SEDUCE | -0.15 (-0.32; 0.01) | 0.07 | 70.5% |

## Sensitivity Analysis in STEMI Patients

**Supplemental Table 7.** Sensitivity analysis using leave-one-out method to assess each study's contribution to the pooled estimates in STEMI

| **1-year target lesion revascularization** | | | |
| --- | --- | --- | --- |
| **Excluded Study** | **Pooled OR (95%CI)** | **P-value** | **I²** |
| REVELATION | 2.08 (0.57- 7.48) | 0.26 | 20.0% |
| Hao et al., 2021 | 2.56 (0.74- 8.88) | 0.13 | 7.7% |
| Wang et al., 2022 | 2.70 (0.81- 8.94) | 0.10 | 1.3% |
| Gobić et al., 2017 | 2.21 (0.70- 7.02) | 0.17 | 15.9% |
| DEB-AMI | 1.20 (0.37- 3.88) | 0.74 | 0.0% |
| **1-year All-cause mortality** | | | |
| **Excluded Study** | **Pooled OR (95%CI)** | **P-value** | **I²** |
| REVELATION | 1.04 (0.23-4.71) | 0.95 | 0.0% |
| Hao et al., 2021 | 0.97 (0.13-6.99) | 0.97 | 0.0% |
| Wang et al., 2022 | 1.05 (0.20-5.37) | 0.95 | 0.0% |
| Gobić et al., 2017 | 1.06 (0.23-4.78) | 0.93 | 0.0% |
| **1-year Cardiovascular mortality** | | | |
| **Excluded Study** | **Pooled OR (95%CI)** | **P-value** | **I²** |
| REVELATION | 0.82 (0.19-3.54) | 0.79 | 0.0% |
| Hao et al., 2021 | 0.67 (0.10-4.30) | 0.67 | 0.0% |
| Wang et al., 2022 | 1.04 (0.23-4.70) | 0.95 | 0.0% |
| Gobić et al., 2017 | 0.83 (0.19-3.55) | 0.81 | 0.0% |
| DEB-AMI | 0.83 (0.21-3.31) | 0.80 | 0.0% |
| **1-year Myocardial infarction** | | | |
| **Excluded Study** | **Pooled OR (95%CI)** | **P-value** | **I²** |
| REVELATION | 1.19 (0.43-3.27) | 0.73 | 0.0% |
| Hao et al., 2021 | 1.20 (0.39-3.69) | 0.74 | 0.0% |
| Wang et al., 2022 | 1.29 (0.38-4.42) | 0.67 | 0.0% |
| Gobić et al., 2017 | 1.28 (0.41-3.94) | 0.66 | 0.0% |
| DEB-AMI | 0.99 (0.35-02.80) | 0.99 | 0.0% |
| **1-year Thrombosis** | | | |
| **Excluded Study** | **Pooled OR (95%CI)** | **P-value** | **I²** |
| Hao et al., 2021 | 1.51 (0.28-8.14) | 0.62 | 0.0% |
| Gobić et al., 2017 | 1.42 (0.10-19.17) | 0.78 | 26.7% |
| DEB-AMI | 0.69 (0.12-3.83) | 0.67 | 0.0% |
| **6-month Late lumen loss** | | | |
| **Excluded Study** | **Pooled SMD (95%CI)** | **P-value** | **I²** |
| REVELATION | -0.23 (-1.59; 1.12) | 0.73 | 96.2% |
| Wang et al., 2022 | -0.21 (-1.57; 1.15) | 0.76 | 96.2% |
| Gobić et al., 2017 | 0.20 (-0.48; 0.89) | 0.55 | 90.1% |
| DEB-AMI | -0.56 (-1.43; 0.29) | 0.19 | 91.2% |

**Supplemental Figure 17.** Sensitivity Analysis of SVD based on DCB ARC statement definition of SVD


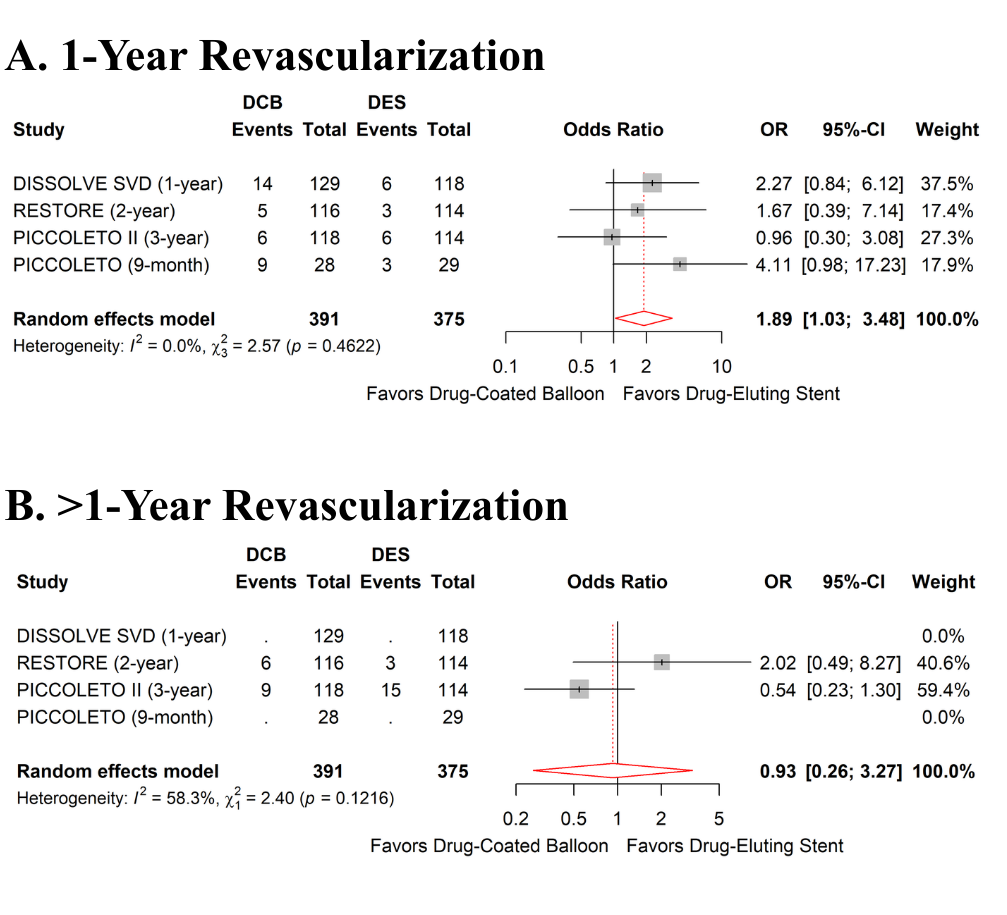


## Sensitivity Analysis in SVD Patients

**Supplemental Table 8.** Sensitivity analysis using leave-one-out method to assess each study's contribution to the pooled estimates in SVD

| **1-year Target lesion revascularization** | | | |
| --- | --- | --- | --- |
| **Excluded Study** | **Pooled OR (95%CI)** | **P-value** | **I²** |
| DISSOLVE SVD | 1.21 (0.67-2.18) | 0.50 | 28.8% |
| RESTORE | 1.34 (0.73-2.46) | 0.33 | 41.6% |
| PICCOLETO II | 1.47 (0.79-2.72) | 0.21 | 39.1% |
| BASKET-SMALL II | 1.41 (0.70-2.85) | 0.33 | 42.2% |
| BELLO | 1.62 (1.01-2.59) | 0.04 | 0.0% |
| PICCOLETO | 1.20 (0.74-1.93) | 0.44 | 8.8% |
| **>1-year Target lesion revascularization** | | | |
| **Excluded Study** | **Pooled OR (95%CI)** | **P-value** | **I²** |
| RESTORE (2-year) | 0.78 (0.48-1.26) | 0.31 | 18.2% |
| PICCOLETO II (3-year) | 0.98 (0.56-1.69) | 0.94 | 18.5% |
| BASKET-SMALL II (3-year) | 0.71 (0.34-1.47) | 0.36 | 27.7% |
| BELLO (2-year) | 0.94 (0.53-1.66) | 0.84 | 29% |
| **1-year All-cause mortality** | | | |
| **Excluded Study** | **Pooled OR (95%CI)** | **P-value** | **I²** |
| DISSOLVE SVD | 0.55 (0.27-1.15) | 0.11 | 0.0% |
| RESTORE | 0.56 (0.27-1.15) | 0.11 | 0.0% |
| PICCOLETO II | 0.59 (0.28-1.21) | 0.15 | 0.0% |
| BASKET-SMALL II | 0.80 (0.20-3.13) | 0.75 | 0.0% |
| BELLO | 0.55 (0.26-1.14) | 0.11 | 0.0% |
| PICCOLETO | 0.55 (0.26-1.14) | 0.11 | 0.0% |
| **>1-year All-cause mortality** | | | |
| **Excluded Study** | **Pooled OR (95%CI)** | **P-value** | **I²** |
| RESTORE (2-year) | 0.92 (0.55-1.52) | 0.75 | 0.0% |
| PICCOLETO II (3-year) | 0.91 (0.54-1.55) | 0.75 | 0.0% |
| BASKET-SMALL II (3-year) | 0.84 (0.27-2.57) | 0.76 | 0.0% |
| BELLO (2-year) | 0.94 (0.57-1.56) | 0.83 | 0.0% |
| **1-year Cardiovascular mortality** | | | |
| **Excluded Study** | **Pooled OR (95%CI)** | **P-value** | **I²** |
| DISSOLVE SVD | 0.49 (0.19-1.24) | 0.13 | 0.0% |
| RESTORE | 0.45 (0.18-1.14) | 0.09 | 0.0% |
| PICCOLETO II | 0.45 (0.18-1.14) | 0.09 | 0.0% |
| BASKET-SMALL II | 0.72 (0.13-3.86) | 0.70 | 0.0% |
| BELLO | 0.45 (0.18-1.13) | 0.09 | 0.0% |
| PICCOLETO | 0.45 (0.18-1.13) | 0.09 | 0.0% |
| **>1-year Cardiovascular mortality** | | | |
| **Excluded Study** | **Pooled OR (95%CI)** | **P-value** | **I²** |
| RESTORE (2-year) | 0.81 (0.40-1.62) | 0.55 | 0.0% |
| PICCOLETO II (3-year) | 0.72 (0.35-1.46) | 0.36 | 0.0% |
| BASKET-SMALL II (3-year) | 1.02 (0.18-5.76) | 0.98 | 0.0% |
| BELLO (2-year) | 0.77 (0.38-1.54) | 0.46 | 0.0% |
| **1-year Myocardial infarction** | | | |
| **Excluded Study** | **Pooled OR (95%CI)** | **P-value** | **I²** |
| DISSOLVE SVD | 1.00 (0.32-3.11) | 0.98 | 39.3% |
| RESTORE | 0.90 (0.29-2.77) | 0.85 | 36.5% |
| PICCOLETO II | 1.27 (0.41-3.95) | 0.66 | 22.9% |
| BASKET-SMALL II | 0.58 (0.19-1.76) | 0.33 | 0.0% |
| BELLO | 1.59 (0.73-3.48) | 0.23 | 0.0% |
| PICCOLETO | 0.89 (0.29-2.74) | 0.85 | 36.0% |
| **>1-year Myocardial infarction** | | | |
| **Excluded Study** | **Pooled OR (95%CI)** | **P-value** | **I²** |
| RESTORE (2-year) | 0.59 (0.21-1.65) | 0.31 | 58.1% |
| PICCOLETO II (3-year) | 0.88 (0.41-1.86) | 0.74 | 19.2% |
| BASKET-SMALL II (3-year) | 0.36 (0.13-0.95) | 0.05 | 0.0% |
| BELLO (2-year) | 0.79 (0.29-2.11) | 0.63 | 33.9% |
| **1-year Thrombosis** | | | |
| **Excluded Study** | **Pooled OR (95%CI)** | **P-value** | **I²** |
| DISSOLVE SVD | 1.10 (0.29-4.09) | 0.88 | 0.0% |
| RESTORE | 1.09 (0.29-4.06) | 0.89 | 0.0% |
| PICCOLETO II | 1.53 (0.39-5.99) | 0.53 | 0.0% |
| BASKET-SMALL II | 0.54 (0.08-3.36) | 0.51 | 0.0% |
| PICCOLETO | 1.08 (0.29-4.03) | 0.89 | 0.0% |
| **>1-year Thrombosis** | | | |
| **Excluded Study** | **Pooled OR (95%CI)** | **P-value** | **I²** |
| RESTORE (2-year) | 0.70 (0.02-18.31) | 0.83 | 74.2% |
| PICCOLETO II (3-year) | 2.54 (0.57-11.25) | 0.21 | 0.0% |
| BASKET-SMALL II (3-year) | 0.23 (0.02-2.43) | 0.22 | 0.0% |
| **6-month Late lumen loss** | | | |
| **Excluded Study** | **Pooled SMD (95%CI)** | **P-value** | **I²** |
| DISSOLVE SVD | -0.44 (-0.6; -0.25) | <0.001 | 0.0% |
| PICCOLETO II | -0.36 (-0.61; -0.12) | <0.001 | 38.9% |
| BELLO | -0.32 (-0.50; -0.14) | <0.001 | 0.0% |

# Meta-Regression Analysis

**Supplemental Table 9**. Meta-regression analysis of comorbidities in patients with ISR (DCB vs DES)

| **Outcome** | **Variable** | **1-year results** | | | | | | **>1-year results** | | | | | |
| --- | --- | --- | --- | --- | --- | --- | --- | --- | --- | --- | --- | --- | --- |
|  |  | **Number of included studies** | **Slope estimate** | **P-value** | **CI lower bound** | **CI upper bound** | **I^2^** | **Number of included studies** | **Slope estimate** | **P-value** | **CI lower bound** | **CI upper bound** | **I^2^** |
| Target lesion revascularization | Age | 10 | 0.0656 | 0.611 | -0.1875 | 0.3186 | 0.00% | 7 | 0.033 | 0.7847 | -0.2054 | 0.2719 | 0.00% |
|  | Male Gender | 10 | -0.0606 | 0.1159 | -0.0149 | 0.1362 | 22.53% | **7** | ***0.0661** | **0.0240** | **0.0087** | **0.1234** | **0.00%** |
|  | Ejection fraction | 5 | 0.1206 | 0.1189 | -0.0310 | 0.2721 | 32.6% | 4 | 0.0842 | 0.1713 | -0.0364 | 0.2069 | 37.3% |
|  | Hypertension | 9 | -0.0102 | 0.6879 | -0.0601 | 0.03597 | 0.00% | 6 | -0.0232 | 0.4032 | -0.0775 | 0.0312 | 0.00% |
|  | ***Diabetes** | **10** | **0.0699** | **0.0028** | **0.0240** | **0.1159** | **0.00%** | 7 | 0.0482 | 0.0845 | -0.0066 | 0.1030 | 55.97% |
|  | Current smoking | 5 | 0.0313 | 0.6552 | -0.1060 | 0.1685 | 0.00% | 4 | 0.0543 | 0.3736 | -0.0653 | 0.1739 | 0.00% |
|  | Dyslipidemia | 9 | -0.0016 | 0.9013 | -0.0276 | 0.0243 | 0.00% | 6 | 0.0009 | 0.9455 | -0.02060 | 0.0279 | 0.00% |
| All-cause mortality | Age | 9 | 0.0199 | 0.9375 | -0.4784 | 0.5183 | 0.00% | 6 | 0.0496 | 0.8277 | -0.3974 | 0.4966 | 0.00% |
|  | Male Gender | 9 | 0.0460 | 0.4392 | -0.0706 | 0.1627 | 0.00% | 6 | 0.0375 | 0.3504 | -0.0412 | 0.1162 | 0.38% |
|  | Ejection fraction | 5 | -0.0713 | 0.6791 | -0.4089 | 0.2664 | 0.00% | 4 | -0.0723 | 0.6129 | -0.3524 | 0.2078 | 0.00% |
|  | Hypertension | 8 | -0.0001 | 0.9988 | -0.01382 | 0.1380 | 0.00% | 5 | 0.0440 | 0.6806 | -0.1655 | 0.2536 | 0.00% |
|  | Diabetes | 9 | -0.292 | 0.4641 | -0.1073 | 0.0489 | 0.00% | 6 | -0.0309 | 0.3934 | -0.1018 | 0.0400 | 0.00% |
|  | Current smoking | 4 | 0.0245 | 0.8319 | -0.2019 | 0.2509 | 0.00% | 3 | 0.0380 | 0.6266 | -0.1152 | 0.1913 | 0.00% |
|  | Dyslipidemia | 8 | 0.0166 | 0.5797 | -0.0421 | 0.0752 | 0.00% | 5 | 0.0404 | 0.2871 | -0.0340 | 0.1147 | 0.00% |
| Cardiovascular Mortality | Age | 10 | -0.1590 | 0.5654 | -0.7009 | 0.3830 | 0.00% | 7 | 0.0188 | 0.9158 | -0.3300 | 0.3676 | 0.00% |
|  | Male Gender | 10 | 0.00571 | 0.3947 | -0.0745 | 0.1887 | 0.00% | 7 | 0.0077 | 0.8578 | -0.0763 | 0.0917 | 0.00% |
|  | Ejection fraction | 5 | -0.0280 | 0.8634 | -0.3467 | 0.2907 | 0.00% | 4 | -0.0491 | 0.5681 | -0.2179 | 0.1196 | 0.00% |
|  | Hypertension | 9 | 0.0237 | 0.6408 | -0.0758 | 0.1232 | 0.00% | 6 | 0.0371 | 0.5068 | -0.0725 | 0.1467 | 0.00% |
|  | Diabetes | 10 | -0.0474 | 0.3071 | -0.1385 | 0.0436 | 0.00% | 7 | -0.0260 | 0.4842 | -0.0989 | 0.0469 | 0.00% |
|  | Current smoking | 5 | 0.0174 | 0.8535 | -0.1451 | 0.1800 | 0.00% | 4 | 0.0078 | 0.8955 | -0.1090 | 0.1247 | 0.00% |
|  | Dyslipidemia | 9 | 0.0178 | 0.6038 | -0.0494 | 0.0851 | 0.00% | 6 | 0.0252 | 0.4535 | -0.0408 | 0.0913 | 0.00% |
| Myocardial Infarction | Age | 10 | 0.0870 | 0.4987 | -0.1646 | 0.3386 | 0.00% | 7 | 0.0971 | 0.4064 | -0.1322 | 0.3264 | 0.00% |
|  | Male Gender | 10 | 0.0075 | 0.8796 | -0.0899 | 0.1050 | 0.00% | 7 | -0.0159 | 0.6868 | -0.0932 | 0.0614 | 0.00% |
|  | Ejection fraction | 5 | -0.0627 | 0.6110 | -0.3044 | 0.1790 | 0.00% | 4 | -0.0382 | 0.6597 | -0.2081 | 0.1317 | 0.00% |
|  | Hypertension | 9 | 0.0135 | 0.6365 | -0.0424 | 0.0694 | 0.00% | 6 | -0.0145 | 0.6167 | -0.0711 | 0.0422 | 0.00% |
|  | Diabetes | 10 | 0.0236 | 0.5233 | -0.0489 | 0.0962 | 0.00% | 7 | 0.0462 | 0.1954 | -0.0237 | 0.1161 | 0.00% |
|  | Current smoking | 5 | 0.0284 | 0.6191 | -0.0835 | 0.1402 | 0.00% | 4 | -0.0226 | 0.6553 | -0.1219 | -.0767 | 0.00% |
|  | Dyslipidemia | 9 | 0.0108 | 0.4783 | -0.0191 | 0.0408 | 0.00% | 6 | 0.0135 | 0.3783 | -0.0165 | 0.0434 | 0.00% |
| Thrombosis | Age | 8 | -0.1758 | 0.5080 | -0.6961 | 0.3446 | 0.00% | 4 | -0.4358 | 0.3606 | -1.3702 | 0.4986 | 0.00% |
|  | Male Gender | 8 | -0.0180 | 0.8223 | -0.1749 | 0.1390 | 0.00% | 4 | 0.0738 | 0.5203 | -0.1511 | 0.2987 | 0.00% |
|  | Ejection fraction | 4 | -0.0988 | 0.5628 | -0.4334 | 0.2358 | 0.00% | 1 | Not applicable | | | | |
|  | Hypertension | 7 | -0.0370 | 0.5108 | -0.1473 | 0.0733 | 0.00% | 4 | -0.0853 | 0.2415 | -0.2279 | 0.0574 | 0.00% |
|  | Diabetes | 8 | 0.0359 | 0.4773 | -0.0631 | 0.1349 | 0.00% | 4 | 0.0985 | 0.2957 | -0.0862 | 0.2832 | 0.00% |
|  | Current smoking | 4 | -0.0975 | 0.2777 | -0.2735 | 0.0785 | 0.00% | 4 | -0.0975 | 0.2777 | -0.2735 | 0.0785 | 0.00% |
|  | Dyslipidemia | 7 | -0.0187 | 0.5320 | -0.0772 | 0.0399 | 0.00% | 4 | -0.1705 | 0.2154 | -0.4402 | 0.0992 | 0.00% |
| 6-month Late lumen loss | Age | 10 | 0.0044 | 0.9364 | -0.1030 | 0.1118 | 0.00% |  | | | | | |
|  | ***Male Gender** | **10** | **0.0341** | **0.0006** | **0.0146** | **0.0536** | **71.61%** |  |  |  |  |  |  |
|  | Ejection fraction | 5 | 0.0417 | 0.0916 | -0.0068 | 0.0902 | 36.95% |  |  |  |  |  |  |
|  | Hypertension | 9 | -0.0146 | 0.1415 | -0.0340 | 0.0049 | 0.36% |  |  |  |  |  |  |
|  | Diabetes | 10 | 0.0003 | 0.9791 | -0.0197 | 0.0202 | 0.00% |  |  |  |  |  |  |
|  | Current smoking | 5 | 0.0150 | 0.4742 | -0.0261 | 0.0561 | 0.00% |  |  |  |  |  |  |
|  | Dyslipidemia | 9 | 0.0040 | 0.4660 | -0.0067 | 0.0147 | 0.00% |  |  |  |  |  |  |

* P-value of <0.05 was considered statistically significant and bolded.

**Supplemental Table 10.** Meta-regression analysis of comorbidities in patients with STEMI (DCB vs DES)

| **Outcome** | **Variable** | **Number of included studies** | **Slope estimate** | **P-value** | **CI lower bound** | **CI upper bound** | **I^2^** |
| --- | --- | --- | --- | --- | --- | --- | --- |
| Target lesion revascularization | Age | 5 | 0.1445 | 0.3237 | -0.1425 | 0.4314 | 0.00% |
|  | Male Gender | 5 | -0.0283 | 0.7657 | -0.2145 | 0.1579 | 0.00% |
|  | Hypertension | 5 | -0.0182 | 0.5394 | -0.0762 | 0.0399 | 0.00% |
|  | Diabetes | 5 | -0.0205 | 0.2250 | -0.0537 | 0.0126 | 0.00% |
|  | History of smoking | 3 | -0.0264 | 0.6765 | -0.1506 | 0.0977 | 0.00% |
|  | Current smoking | 3 | -0.0434 | 0.3355 | -0.1317 | 0.0449 | 0.00% |
|  | Dyslipidemia | 4 | -0.0189 | 0.5838 | -0.0866 | 0.0488 | 0.00% |
| All-cause mortality | Age | 4 | 0.0098 | 0.9629 | -0.4032 | 0. 4228 | 0.00% |
|  | Male Gender | 4 | 0. 0005 | 0.9959 | -0.2059 | 0.2070 | 0.00% |
|  | Hypertension | 4 | -0.0017 | 0.9638 | -0.0737 | 0.0704 | 0.00% |
|  | Diabetes | 4 | 0.0001 | 0.9967 | -0.0526 | 0.0528 | 0.00% |
|  | History of smoking | 3 | -0.0026 | 0.9747 | -0.1640 | 0.1588 | 0.00% |
|  | Dyslipidemia | 3 | 0.0010 | 0.9796 | -0.0786 | 0.0807 | 0.00% |
| Cardiovascular mortality | Age | 5 | 0.1444 | 0.5251 | -0.3008 | 0.5895 | 0.00% |
|  | Male Gender | 5 | -0.0532 | 0.6381 | -0.2751 | 0.1686 | 0.00% |
|  | Hypertension | 5 | -0.0262 | 0.5205 | -0.1061 | 0.0537 | 0.00% |
|  | Diabetes | 5 | -0.0144 | 0.5967 | -0.0676 | 0.0388 | 0.00% |
|  | History of smoking | 3 | -0.0026 | 0.9747 | -0.1640 | 0.1588 | 0.00% |
|  | Current smoking | 3 | -0.0307 | 0.6133 | -0.1497 | 0.0883 | 0.00% |
|  | Dyslipidemia | 4 | -0.0222 | 0.6087 | -0.1074 | 0.0629 | 0.00% |
| Myocardial infarction | Age | 5 | 0.0506 | 0.7131 | -0.2192 | 0.3205 | 0.00% |
|  | Male Gender | 5 | 0.0009 | 0.9886 | -0.1160 | 0.1177 | 0.00% |
|  | Hypertension | 5 | -0.0056 | 0.8200 | -0.0537 | 0.0425 | 0.00% |
|  | Diabetes | 5 | -0.0049 | 0.7492 | -0.0349 | 0.0251 | 0.00% |
|  | History of smoking | 3 | -0.0059 | 0.9212 | -0.1238 | 0.1119 | 0.00% |
|  | Current smoking | 3 | -0.0270 | 0.5239 | -0.1098 | 0.0559 | 0.00% |
|  | Dyslipidemia | 4 | -0.0030 | 0.9029 | -0.0511 | 0.0451 | 0.00% |
| Thrombosis | Age | 3 | 0.3326 | 0.6964 | -1.3380 | 2.0032 | 0.00% |
|  | Male Gender | 3 | 0.0652 | 0.7151 | -0.2847 | 0.4150 | 0.00% |
|  | Hypertension | 3 | 0.1521 | 0.5151 | -0.3058 | 0.6099 | 0.00% |
|  | Diabetes | 3 | -0.0625 | 0.3868 | -0.2040 | 0.0790 | 0.00% |
|  | History of smoking | 3 | -0.0264 | 0.6765 | -0.1506 | 0.0977 | 0.00% |
|  | Dyslipidemia | 4 | -0.0189 | 0.5838 | -0.0866 | 0.0488 | 0.00% |
| 6-month Late lumen loss | Age | 4 | 0.0586 | 0.7124 | -0.2530 | 0.3702 | 0.00% |
|  | Male Gender | 4 | 0.0587 | 0.3235 | -0.0579 | 0.1754 | 0.00% |
|  | Hypertension | 4 | -0.0005 | 0.9881 | -0.0632 | 0.0622 | 0.00% |
|  | Diabetes | 4 | -0.0003 | 0.9871 | -0.0336 | 0.0331 | 0.00% |
|  | History of smoking | 3 | -0.0059 | 0.9212 | -0.1238 | 0.1119 | 0.00% |
|  | Current smoking | 3 | -0.0140 | 0.5925 | -0.0655 | 0.0374 | 0.00% |
|  | Dyslipidemia | 4 | 0.0102 | 0.7065 | -0.0427 | 0.0630 | 0.00% |

**Supplemental Table 11**. Meta-regression analysis of comorbidities in patients with SVD (DCB vs DES)

| **Outcome** | **Variable** | **1-year results** | | | | | | **>1-year results** | | | | | |
| --- | --- | --- | --- | --- | --- | --- | --- | --- | --- | --- | --- | --- | --- |
|  |  | **Number of included studies** | **Slope estimate** | **P-value** | **CI lower bound** | **CI upper bound** | **I^2^** | **Number of included studies** | **Slope estimate** | **P-value** | **CI lower bound** | **CI upper bound** | **I^2^** |
| Target lesion revascularization | Age | 6 | -0.0421 | 0.6654 | -0.2327 | 0.1486 | 0.00% | 4 | -0.0805 | 0.5579 | -0.3496 | 0.1887 | 0.00% |
|  | Male Gender | 6 | -0.0964 | 0.3353 | -0.2925 | 0.0997 | 0.00% | 4 | -0.1276 | 0.2576 | -0.3485 | 0.0933 | 0.00% |
|  | Ejection fraction | 4 | 0.2166 | 0.2540 | -0.1556 | 0.5889 | 0.00% | 3 | 0.3782 | 0.1301 | -0.1115 | 0.8680 | 0.00% |
|  | Hypertension | 6 | -0.0226 | 0.5807 | -0.1030 | 0.0577 | 0.00% | 4 | 0.0141 | 0.6895 | -0.0551 | 0.0834 | 0.00% |
|  | Diabetes | 6 | 0.0179 | 0.8517 | -0.1697 | 0.2055 | 0.00% | 4 | -0.0167 | 0.8650 | -0.2090 | 0.1756 | 0.00% |
|  | Current smoking | 5 | 0.0812 | 0.1279 | -0.0233 | 0.1858 | 0.00% | 4 | 0.0915 | 0.0797 | -0.0108 | 0.1939 | 0.00% |
|  | dyslipidemia | 6 | -0.0208 | 0.2491 | -0.0563 | 0.0146 | 0.00% | 4 | 0.0011 | 0.9532 | -0.0371 | 0.0394 | 0.00% |
| All-cause mortality | Age | 6 | -0.0708 | 0.6694 | -0.3960 | 0.2543 | 0.00% | 4 | 0.0092 | 0.9578 | -0.3297 | 0.3480 | 0.00% |
|  | Male Gender | 6 | 0.0827 | 0.6998 | -0.3377 | 0.5032 | 0.00% | 4 | -0.1139 | 0.6259 | -0.5718 | 0.3440 | 0.00% |
|  | Ejection fraction | 4 | 0.2698 | 0.6080 | -0.7612 | 1.3009 | 0.00% | 3 | -0.0088 | 0.9817 | -0.7585 | 0.7409 | 0.00% |
|  | Hypertension | 6 | -0.0193 | 0.7336 | -0.1304 | 0.0918 | 0.00% | 4 | 0.0002 | 0.9965 | -0.0695 | 0.0699 | 0.00% |
|  | Diabetes | 6 | 0.0826 | 0.4930 | -0.1536 | 0.3188 | 0.00% | 4 | -0.0335 | 0.7756 | -0.2637 | 0.1968 | 0.00% |
|  | Current smoking | Not applicable | | | | | | 5 | 0.0023 | 0.9888 | -0.3175 | 0.3221 | 0.00% |
|  | dyslipidemia | 6 | -0.0019 | 0.9589 | -0.0747 | 0.0709 | 0.00% | 4 | -0.0037 | 0.8688 | -0.0479 | 0.0405 | 0.00% |
| Cardiovascular mortality | Age | 6 | -0.0296 | 0.8744 | -0.3970 | 0.3378 | 0.00% | 4 | 0.0345 | 0.8740 | -0.3919 | 0.4609 | 0.00% |
|  | Male Gender | 6 | 0.1390 | 0.6264 | -0.4206 | 0.6986 | 0.00% | 4 | 0.1043 | 0.7742 | -0.6082 | 0.8168 | 0.00% |
|  | Ejection fraction | 4 | -0.2426 | 0.6966 | -1.4623 | 0.9771 | 0.00% | 3 | -0.5241 | 0.4097 | -1.7701 | 0.7219 | 0.00% |
|  | Hypertension | 6 | -0.0399 | 0.5656 | -0.1758 | 0.0961 | 0.00% | 4 | -0.0242 | 0.6639 | -0.1332 | 0.0849 | 0.00% |
|  | Diabetes | 6 | 0.1083 | 0.4857 | -0.1961 | 0.4126 | 0.00% | 4 | 0.0013 | 0.9937 | -0.3203 | 0.3229 | 0.00% |
|  | Current smoking | Not applicable | | | | | | 5 | 0.0023 | 0.9888 | -0.3175 | 0.3221 | 0.00% |
|  | dyslipidemia | Not applicable | | | | | | 4 | -0.0168 | 0.6447 | -0.0882 | 0.0546 | 0.00% |
| Myocardial infarction | Age | 6 | 0.0501 | 0.8216 | -0.3857 | 0.4860 | 0.00% | 4 | 0.1162 | 0.6031 | -0.3217 | 0.5540 | 0.00% |
|  | Male Gender | 6 | -0.2650 | 0.1940 | -0.6649 | 0.1349 | 0.00% | 4 | -0.1370 | 0.4698 | -0.5086 | 0.2346 | 0.00% |
|  | Ejection fraction | 4 | 0.5061 | 0.2298 | -0.3200 | 1.3322 | 0.00% | 3 | 0.7416 | 0.0856 | -0.1040 | 1.5871 | 0.00% |
|  | Hypertension | 6 | 0.0346 | 0.5919 | -0.0919 | 0.1610 | 0.00% | 4 | 0.0709 | 0.0728 | -0.0065 | 0.1483 | 0.00% |
|  | Diabetes | 6 | -0.1282 | 0.3737 | -0.4106 | 0.1542 | 0.00% | 4 | -0.1571 | 0.0882 | -0.3377 | 0.0235 | 0.00% |
|  | Current smoking | 5 | 0.2178 | 0.0543 | -0.0040 | 0.4396 | 0.00% | Not applicable | | | | | |
|  | dyslipidemia | 6 | -0.0083 | 0.8325 | -0.0854 | 0.0688 | 0.00% | Not applicable | | | | | |
| Thrombosis | Age | 5 | 0.1132 | 0.6137 | -0.3261 | 0.5525 | 0.00% | 3 | 0.1446 | 0.7681 | -0.8164 | 1.1055 | 0.00% |
|  | Male Gender | 5 | 0.0446 | 0.9176 | -0.8013 | 0.8906 | 0.00% | 3 | 0.0331 | 0.9868 | -3.8812 | 3.9475 | 0.00% |
|  | Ejection fraction | 4 | 0.4978 | 0.4126 | -0.6930 | 1.6885 | 0.00% | 3 | 1.5185 | 0.0642 | -0.0897 | 3.1266 | 0.00% |
|  | Hypertension | 5 | 0.0893 | 0.2340 | -0.0577 | 0.2362 | 0.00% | 3 | 0.1445 | 0.0597 | -0.0059 | 0.2948 | 0.00% |
|  | Diabetes | 5 | -0.1134 | 0.5680 | -0.5027 | 0.2759 | 0.00% | 3 | -0.1939 | 0.6584 | -1.0531 | 0.6654 | 0.00% |
|  | Current smoking |  |  |  |  |  | 0.00% | 5 | 0.0023 | 0.9888 | -0.3175 | 0.3221 | 0.00% |
|  | dyslipidemia | 5 | 0.0644 | 0.2084 | -0.0360 | 0.1649 | 0.00% | 3 | 0.0996 | 0.0522 | -0.0009 | 0.2002 | 0.00% |

**Supplemental Table 12.** Meta-regression results for predicting variables and late lumen loss outcomes in the SVD group

| **Outcome** | **Variable** | **Number of included studies** | **Slope estimate** | **P-value** | **CI lower bound** | **CI upper bound** | **I^2^** |
| --- | --- | --- | --- | --- | --- | --- | --- |
| 6-month Late lumen loss | Male Gender | 4 | -0.1370 | 0.4698 | -0.5086 | 0.2346 | 0.00% |
|  | Current smoking | 5 | 0.2178 | 0.0543 | -0.0040 | 0.4396 | 0.00% |

# Publication Bias

## ISR

**Supplemental Figure 18**. Funnel plots for target lesion revascularization in patients with ISR A.1-year target lesion revascularization

B. >1-year target lesion revascularization

**A) 1-year Target Lesion Revascularization**

**B) >1-year Target Lesion Revascularization**







**Supplemental Figure 19.** Funnel plots for all-cause mortality in patients with ISR A.1-year all-cause mortality B. >1-year all-cause mortality

**B) >1-year All-Cause Mortality**

**A) 1-year All-Cause Mortality**







**Supplemental Figure 20**. Funnel plots for cardiovascular mortality in patients with ISR A.1-year cardiovascular mortality B. >1-year cardiovascular mortality

**B) >1-year Cardiovascular Mortality**

**A) 1-year Cardiovascular Mortality**



**

**

**Supplemental Figure 21**. Funnel plots for myocardial infarction in patients with ISR A.1-year myocardial infarction B. >1-year myocardial infarction

**B) >1-year Myocardial Infarction**

**A) 1-year Myocardial Infarction**



**

**

**Supplemental Figure 22.** Forest plots for 1-year Thrombosis in patients with ISR

**A) 1-year Thrombosis**

**B) >1-year Thrombosis**



**

**

**Supplemental Figure 23**. Forest plot for Late lumen loss in patients with ISR

**

**

## STEMI

**Supplemental Figure 24**. Forest plot for Target lesion revascularization in patients with STEMI





P= 0.82 (Egger’s test)

**Supplemental Figure 25.** Forest plot for All-cause mortality in patients with STEMI





P= 0.10 (Egger’s test)

**Supplemental Figure 26.** Forest plot for cardiovascular mortality in patients with STEMI





P= 0.62 (Egger’s test)

**Supplemental Figure 27.** Forest plot for Myocardial Infarction in patients with STEMI





P= 0.37 (Egger’s test)

**Supplemental Figure 28**. Forest plot for Thrombosis in patients with STEMI





P= 0.89 (Egger’s test)

**Supplemental Figure 29**. Forest plot for Late lumen loss in patients with STEMI





P= 0.73 (Egger’s test)

## SVD

**Supplemental Figure 30.** Funnel plots for target lesion revascularization in SVD patients A. 1-year target lesion revascularization

B. >1-year target lesion revascularization

**B) >1-year Target Lesion Revascularization**

**A) 1-year Target Lesion Revascularization**







P= 0.59 (Egger’s test)

P= 0.87 (Egger’s test)

**Supplemental Figure 31.** Funnel plots for all-cause mortality in SVD patients A. 1-year all-cause mortality B. >1-year all-cause mortality

**B) >1-year All-Cause Mortality**

**A) 1-year All-Cause Mortality**







P= 0.18 (Egger’s test)

P= 0.47 (Egger’s test)

**Supplemental Figure 32**. Funnel plots for cardiovascular mortality in SVD patients A. 1-year cardiovascular mortality B. >1-year cardiovascular mortality

**B) >1-year Cardiovascular Mortality**

**A) 1-year Cardiovascular Mortality**







P= 0.81 (Egger’s test)

P= 0.06 (Egger’s test)

**Supplemental Figure 33.** Funnel plots for Myocardial Infarction in SVD patients A. 1-year Myocardial infarction B. >1-year Myocardial infarction

**A) 1-year Myocardial infarction**

**B) >1-year Myocardial infarction**







P= 0.32 (Egger’s test)

P= 0.60 (Egger’s test)

**Supplemental Figure 34.** Funnel plots for thrombosis in SVD patients A. 1-year thrombosis B. >1-year thrombosis

**A) 1-year Thrombosis**

**B) >1-year Thrombosis**







P= 0.51 (Egger’s test)

P= 0.33 (Egger’s test)

**Supplemental Figure 35.** Funnel plots for late lumen loss in SVD patients
